# Untargeted metabolomics analysis on kidney tissues from mice reveals potential hypoxia biomarkers

**DOI:** 10.1038/s41598-023-44629-y

**Published:** 2023-10-16

**Authors:** Muhammad Imran Sajid, Francisco J. Nunez, Farideh Amirrad, Moom Rahman Roosan, Tom Vojtko, Scott McCulloch, Amal Alachkar, Surya M. Nauli

**Affiliations:** 1 Department of Biomedical and Pharmaceutical Sciences, Chapman University, 9401 Jeronimo Road, Irvine, CA 92618-1908 USA; 2 Faculty of Pharmaceutical Sciences, University of Central Punjab, Lahore, 54000 Pakistan; 3 Metabolon Inc, 617 Davis Drive, Suite 100, Morrisville, NC 27560 USA; 4 Department of Pharmaceutical Sciences, University of California, Irvine, CA 92697-4625 USA; 5 Department of Medicine, University of California Irvine, Orange, CA 92868 USA

**Keywords:** Biochemistry, Biological techniques, Computational biology and bioinformatics, Biomarkers, Nephrology

## Abstract

Chronic hypoxia may have a huge impact on the cardiovascular and renal systems. Advancements in microscopy, metabolomics, and bioinformatics provide opportunities to identify new biomarkers. In this study, we aimed at elucidating the metabolic alterations in kidney tissues induced by chronic hypoxia using untargeted metabolomic analyses. Reverse phase ultrahigh performance liquid chromatography-mass spectroscopy/mass spectroscopy (RP–UPLC–MS/MS) and hydrophilic interaction liquid chromatography (HILIC)–UPLC–MS/MS methods with positive and negative ion mode electrospray ionization were used for metabolic profiling. The metabolomic profiling revealed an increase in metabolites related to carnitine synthesis and purine metabolism. Additionally, there was a notable increase in bilirubin. Heme, N-acetyl-l-aspartic acid, thyroxine, and 3-beta-Hydroxy-5-cholestenoate were found to be significantly downregulated. 3-beta-Hydroxy-5-cholestenoate was downregulated more significantly in male than female kidneys. Trichome Staining also showed remarkable kidney fibrosis in mice subjected to chronic hypoxia. Our study offers potential intracellular metabolite signatures for hypoxic kidneys.

## Introduction

It is well known that living under hypoxic conditions has several distressing effects on the kidney^1^ that resulted in the coining of the term "High Altitude Renal Syndrome" (HARS)^2^. The key findings of HARS are chronic kidney disease (CKD), polycythemia (excessive erythrocytosis), hyperuricemia, glomerulomegaly, microalbuminuria, elevated systemic blood pressure, and kidney failure^2^. The kidneys have an abundant blood supply (20%–25% of cardiac output) and high blood flow and are susceptible to the effects of hypoxia^3^. In 1998, Fine et al. proposed the "Chronic Hypoxia Hypothesis" for the pathogenesis of CKD based on the observation that hypoxia drives kidney fibrogenesis and tubulointerstitial injury is characteristic of all progressive renal diseases^4,5^. Furthermore, Shamloo et al. reported that fetal kidneys are more susceptible to hypoxia, possibly due to a “triple-hit hypoxia” phenomenon, which implicates three factors (triple-hit) affecting fetal kidneys in response to hypoxia^6^.

Over the past three decades, substantial evidence has been accumulated concluding that hypoxia is a common cause of both Acute Kidney Injury (AKI) and CKD, that renal tissue hypoxia, at least under in-vitro conditions, drives a signaling cascade that leads to tissue damage and that tissue hypoxia can lead to renal pathology independent of other known risk factors of kidney disease^7^. However, relatively little progress has been made in determining the causative role of hypoxia in kidney disease and if preventing hypoxia can prevent or delay renal disease^7^.

Hypoxia, defined as the deficiency of oxygen in the biotic environment, causes cellular stress and alters normal metabolic activity^8^. Several studies investigated the adaptation of cellular metabolism in response to hypoxia^8^. For instance, it has been reported that exposure to hypoxia causes an increased production of reactive oxygen species that causes damaging effects on a variety of cellular components^9^. Additionally, hypoxia alters several key metabolic processes, including glucose uptake, glycolysis, oxidative metabolism, lipolysis, and lipogenesis in adipocytes^10^. Chen et al. comprehensively reviewed the pathophysiological implications of hypoxia in several diseases and concluded that hypoxia plays critical roles in the pathogenesis of major causes of mortality, including cancer, metabolic diseases, myocardial ischemia, chronic heart and kidney diseases, and in reproductive diseases such as preeclampsia and endometriosis^10^. However, very few studies investigated the metabolic biomarkers of hypoxia.

Metabolomics is a powerful tool to study such complex metabolism by quantitatively analyzing metabolic response to pathological, physical, or chemical stimulus including changes in oxygen availability^11,12^ Metabolomics is an emerging field that investigates the metabolites at a cellular, organ, or organism level to identify the metabolites overexpressed or inhibited in a particular condition or disease^13^. With the technological advancement in chromatography, mass spectrometry, and bioinformatics tools, metabolomics can involve an "untargeted" screening where thousands of metabolites can be screened and profiled to understand the relative differences in diseased conditions or genetic differences^14,15^. Investigating the difference in metabolites in biological samples provides valuable insights into the animal models raised in severe conditions such as hypoxia. For instance, several metabolomics studies have been performed in serum samples, urine, and tissues^16–19^. However, to the best of our knowledge, this is the first report in which we utilized the metabolomic and histological approach to understand the effect of hypoxia on kidney tissues. We used advanced Ultrahigh Performance Liquid Chromatography-Tandem Mass Spectroscopy, bioinformatics tools, and robust statistical analysis to investigate the metabolic alterations observed in response to hypoxia in kidney tissue, which led to the discovery of potential biomarkers for hypoxia. Furthermore, National Institute of Health in its policy published in 2016 emphasized for researchers to include sex as a biological variable in all research designs, analyses, and reporting in vertebrate animal and human studies^20^. Sex- and gender-aware investigations are critical to the conduct of rigorous and transparent science and the advancement of personalized medicine. In the current study, we also investigated and analyzed the metabolic differences in male and female groups in response to hypoxia.

## Materials and methods

### Ethics statement

All animal experiments in the current study were approved by Chapman University Institutional Animal Care and Use Committee (IACUC# 2020-1132 and PHS# D17-00960) and were conducted in accordance with the “Guide for the Care and Use of Laboratory Animals” prepared by Institute for Laboratory Animal Research (ILAR) of the National Research Council in the USA^21^. Furthermore, all methods of animal experiments are reported in accordance with ARRIVE (Animal Research: Reporting of In Vivo Experiments) guidelines^22^.

### Animals and tissue collection

A total of eighteen, 8 weeks old, adult, fertile, male and female wildtype (WT) mice were used in our study. The animals were chosen randomly based on age. Ten mice were exposed to hypoxic chambers (described below) for 6 weeks and the remaining eight mice were raised in the same room at normal room temperature, pressure, and airflow (normoxic conditions). The kidney samples were collected from each animal after euthanasia and immediately frozen in liquid nitrogen. A total of twelve kidney samples (one kidney sample from each of the mice) was used for Masson’s Trichrome Staining (described below). One frozen kidney sample from each of the eighteen mice were sent to Metabolon Inc. for etabolomics profiling, the details including weight of the samples, gender, and the condition (hypoxic vs normoxic) of these samples are provided in Supplementary Information 1 under tab “Sample Meta Data”.

### Masson’s trichrome staining

**Figure 1:**
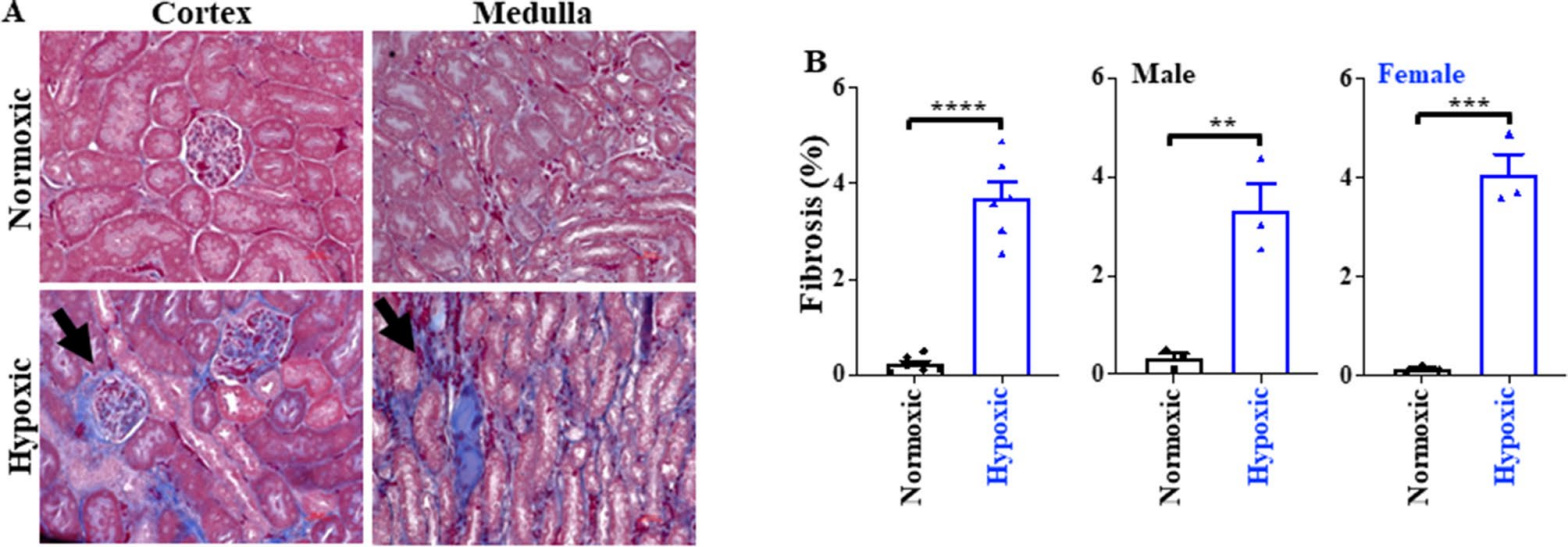
Masson’s trichrome staining. (**A**) Representative images of the cortex and medulla of the kidney under hypoxic and normoxic conditions. (**B**) Bar graphs represent the quantification of fibrosis and illustrate significant fibrosis in male and female mice due to hypoxia. N = 6 for hypoxic kidneys and N = 6 for normoxic kidneys from independent mice. Male mice were 3 each in the normoxic and hypoxic groups; similarly, female mice were 3 each in normoxic and hypoxic groups. **p < 0.01; ***p < 0.001.

To evaluate kidney fibrosis, we used Masson’s trichrome staining^23^. A total of twelve mice kidney samples (one kidney from each mouse) were used for histological study and were divided into two groups (hypoxic and normoxic) containing six mice in each group (see Fig. 1 legend). Also, both normoxic and hypoxic groups consisted of equal number of male and female mice (N = 3). The kidney tissues were collected and fixed in 10% formalin. The tissues were dehydrated in ethanol and xylene, embedded in liquid paraffin, and cut with a thickness of 5 μm. Cut sections were stained with Masson’s trichrome kit (Cat# 25088-1; Polysciences, Inc.), and images were visualized and captured using KEYENCE BZ-X710. The kidney fibrosis was quantified by calculating the percentage of kidney tissue occupied by collagen fibers (blue color) in different kidney sections using a Nikon Eclipse Ti microscope.

### Hypoxia chambers

Eight weeks old, adult, fertile, male and female WT mice (N = 10) were maintained in a normobaric hypoxic chamber (BioSpherix, NY, USA) with 10% O_2_ (corresponds to about 5800-m altitude) and CO_2_ less than 1000 ppm for six consecutive weeks (hypoxic condition). Matching control animals (N = 8) were kept for six consecutive weeks outside the hypoxic chamber in the same room at normal room temperature, pressure, and airflow (normoxic condition). Static Innovive cages were used to allow air/gas exchange from the outside, and these cages were put in the Biospherix chamber (https://sites.chapman.edu/cilia/biospherix/). The chamber had its own O_2_/N_2_/air regulators to adjust the levels of PO_2_ and PCO_2_ during the experiments. O_2_ levels were regulated through N_2_ gas, and CO_2_ level was controlled with soda lime (sodasorb) and an air tank. On the first day of exposure, the O_2_ level in the chamber decreased by 1% every 20 min to up to 10% and remained constant at this level for the duration of the study. In the hypoxic chamber, mice had free access to water and diet, and the disposable cage was changed every week (q7days) to ensure the clean environment for the animals; the cage changing did not take more than a minute per cage. The filter top on the Innovive cage lid provided enough air ventilation in the hypoxic chamber and was acceptable without the forced air ventilation from the rack. The O_2_ and CO_2_ levels in the chamber were controlled by the ProOx P110 and P120PPM, respectively.

After 42 days of hypoxic exposure, animals were removed and euthanized by asphyxiation using a CO_2_ gas chamber for 5 min, followed by cervical dislocation as a secondary method to confirm death. The kidneys were collected from euthanized animals and were immediately frozen in liquid nitrogen for further study.

### Sample accessioning and preparation

The weighed samples were received by Metabolon Inc. in frozen form in liquid nitrogen and were immediately inventoried in Metabolon’s Laboratory Information Management System (LIMS) with a unique identifier, which tracked all the samples throughout the experimental process, data generation, and analysis. All samples were kept frozen at − 80 °C until processed^24,25^. The details of the samples received by the Metabolon Inc. are provided in Supplementary Information 1 under the tab “Sample Meta Data”.

On the day of extraction, the kidney tissues were thawed on ice and the proteins were precipitated using methanol for 2 min under vigorous shaking using Glen Mills GenoGrinder 2000. The samples were centrifuged, followed by placement on a TurboVap® (Zymark) to remove the organic solvent. The sample extracts were stored overnight under nitrogen before preparation for analysis^24,25^. Later, the sample extract was dried, followed by reconstitution with compatible solvents (50 µL of 0.1% formic acid in H_2_O, 50 µL of 6.5 mM ammonium bicarbonate in H_2_O, or 50 µL of 0.1% formic acid in 10% methanol) to make five fractions of each sample. For instance, one aliquot was reconstituted in the reconstitution solvents containing instrument internal standards that were used to monitor instrument performance and as retention index markers. Fixed concentration and volume of the standards were added to ensure the injection and chromatographic consistencies. The sample extract was divided into five fractions. Two separate Reverse Phase-Ultrahigh Performance Liquid Chromatography-Mass Spectroscopy/Mass Spectroscopy (RP)/UPLC–MS/MS) methods with positive ion mode electrospray ionization (ESI) were used on two fractions. Another RP/UPLC–MS/MS with negative ion mode ESI was used on the third fraction, and the fourth fraction was used for analysis by Hydrophilic Interaction Chromatography (HILIC) UPLC–MS/MS with negative ion mode ESI. The fifth fraction was kept as a backup^24,25^.

### Quality assurance and control

Instrumental performance monitoring and precise chromatographic alignment were carried out using several controls run with the experimental samples. These controls included a pooled matrix sample from each experimental sample, human plasma pool, water samples serving as blanks, and Quality Control (QC) standards. Tables S1 and S2 describe these QC samples and standards^24,25^. Experimental samples were randomized with QC samples across the platform run, as outlined in Fig. S1 in Supplementary Information 3.

### UPLC–MS/MS

“Waters Acquity Ultrahigh Performance Liquid Chromatography” was used for all methods along with a high resolution/accurate mass spectrometer (Thermo Scientific Q-Exactive) equipped with a heated ESI source and a mass analyzer with 35,000 mass resolution. Four methods were used to analyze the metabolites. One method used acidic positive ion conditions optimized for hydrophilic compounds, in which the sample aliquot was eluted from a C_18_ column (Waters UPLC BEH C18-2.1 × 100 mm, 1.7 µm) using a gradient of water and methanol supplemented with 0.05% perfluoropentanoic acid (PFPA) and 0.1% formic acid (FA). The second method was optimized for more hydrophobic compounds, in which the sample aliquot eluted from the C_18_ column (Waters UPLC BEH C18-2.1 × 100 mm, 1.7 µm) using a gradient of methanol/acetonitrile/water containing 0.05% PFPA and 0.01% FA. The third sample aliquot was run in basic negative ion conditions and was eluted from a separate dedicated C_18_ column using a gradient of methanol/water with 6.5 mM ammonium bicarbonate (pH = 8). The fourth sample aliquot was run under negative ionization and was eluted from the HILIC column (Waters UPLC BEH Amide 2.1 × 150 mm, 1.7 µm) using a gradient of acetonitrile/water with 10 mM ammonium formate (pH = 10.8). Following UPLC, the MS analysis was performed, where the MS scan range covered 70–1000 m/z^24,25^. Raw data files were archived and extracted for further analysis.

### Bioinformatics and laboratory information management system (LIMS)

The bioinformatics system consisted of four major components: LIMS, the peak-identification software, data processing tools for compound identification, and software for data analysis. A local area network backbone and a database server run on Oracle 10.2.0.1 Enterprise Edition were the foundation for these bioinformatics components^24,25^. The Metabolon’s LIMS was enabled for complete auditable laboratory automation that covers sample accessioning, preparation, instrumental analysis, reporting, and advanced data analysis^24,25^.

### Data extraction and compound identification

Following analysis, the raw data was extracted, and the compounds were identified using library entries of purified standards. Metabolon Inc. maintains a compound library containing information on the retention time/index (RI), chromatographic data, MS/MS spectral data, and the mass-to-charge ratio. Over 3300 commercially available purified standard compounds have been registered into LIMS. Furthermore, biochemical identifications were performed using retention index within a narrow RI window, accurate mass match (± 10 ppm), and the MS/MS forward and reverse scores. The details of these processes are described in reports by Evan et al. and Ford et al.^24,25^.

### Biomarker discovery

The biomarker discovery feature of MetaboAnalyst 5.0 was used for biomarker discovery that provides the receiver operating characteristic (ROC) curve-based approach for identifying potential biomarkers. Xia et al. provided a comprehensive tutorial on translational biomarker discovery in clinical metabolomics^26^. Briefly, the ROC curve is the plot of the true positive rate (TPR) (also known as sensitivity) against the false positive rate (FPR) (also known as specificity) at various threshold settings. ROC curves are summarized into a single metric called Area under the curve (AUC), which represents the probability that a diagnostic test or a classifier will rank a randomly chosen positive instance higher than a randomly chosen negative one. If all positive samples are ranked before negative ones (i.e., a perfect classifier), the AUC is 1.0. A rough guide for assessing the utility of a biomarker based on its AUC is as follows: 0.9–1.0 = excellent; 0.8–0.9 = good; 0.7–0.8 = fair; 0.6–0.7 = poor; 0.5–0.6 = fail^26^. We selected the metabolites with an AUC > 0.8.

### Data analyses

Comprehensive information on the classification, physical and chemical properties of the metabolites detected in this study can be obtained from Human Metabolome Database (HMDB; https://www.hmdb.ca/, KEGG: Kyoto Encyclopedia of Genes and Genomes; (https://www.genome.jp/kegg/), and SMPDB: The Small Molecule Pathway Database (https://www.smpdb.ca/). We used log-transformed and normalized data for statistical analysis using MetaboAnalyst 5.0 (https://www.metaboanalyst.ca/), which is a comprehensive web-based tool dedicated to metabolomic data analysis, and GraphPad Prism Version 9.5.0 (730). (The complete dataset is available in Supplementary Infromation 1). Briefly, Volcano plot analysis was performed to analyze the significant differences in metabolites between hypoxic and normoxic groups; p-values were calculated using student's t-test, and fold-change (FC) of metabolites between hypoxic vs. normoxic group was calculated. The differential metabolites between the groups were identified by a p-value cut-off of < 0.05 and an FC > 2.0.

Most omics experimental design aims to compare samples from a control (e.g., diseases or treatment)^27^. Predictive models such as orthogonal partial least discriminant analysis (OPLS-DA) are widely used for discriminant analysis^28,29^ and have been demonstrated as a powerful tool with easier interpretation of the qualitative data analysis^30,31^. We used OPLS-DA to observe the visible separation of metabolites between the groups. The significant metabolites from OPLS-DA were selected based on the variable importance in projection (VIP) > 1, as reported earlier by Hasegawa et al.^32^, and used for Pathway Analysis from MetaboAnalyst 5.0., using *Mus. musculus* library containing 82 pathways.

Hierarchical clustering of the data was done using healtmap^33^. A heatmap provides an intuitive visualization of the data, where each colored cell on the map corresponds to the log-normalized concentration of the metabolites in the data. The rows in the heatmap represent individual metabolites, and the columns represent biological replicates. Distance was measured using Euclidean Correlations and the Ward clustering algorithm. For analyzing the interaction of gender with hypoxic state, we used the multivariate analysis feature from MetaboAnalyst 5.0^34^, the study design of 'two-factor independent samples' was set, and two-way ANOVA analysis was performed to identify differences and interactions between gender and condition (hypoxic vs. normoxic).

### Patents

The study results are being considered for "invention and discovery disclosure" at Chapman University.

## Results

### Masson’s trichrome staining

Masson's trichrome staining was used to investigate the effect of hypoxia on kidney tissues. Normoxic kidneys (N = 6) served as negative control, and the hypoxic kidneys (N = 6) were used as the experimental group. The histological images clearly demonstrated significant fibrosis in the cortex and medulla of the kidney. Figure 1 shows the representative images of the cortex and medullary kidneys from the mice raised under normoxic and hypoxic conditions. Figure 1A visibly demonstrates the effect of hypoxia on the kidney tissues.

Furthermore, renal fibrosis was quantified both in male and female mice by calculating the percentage of renal tissue occupied with the collagen fibers (blue color) in different kidney sections. The results indicated significant fibrosis in the hypoxic group in both genders. Figure 1B represents the % fibrosis in the hypoxic vs. normoxic group.

### Metabolic profiling of mouse kidney tissues

**Figure 2:**
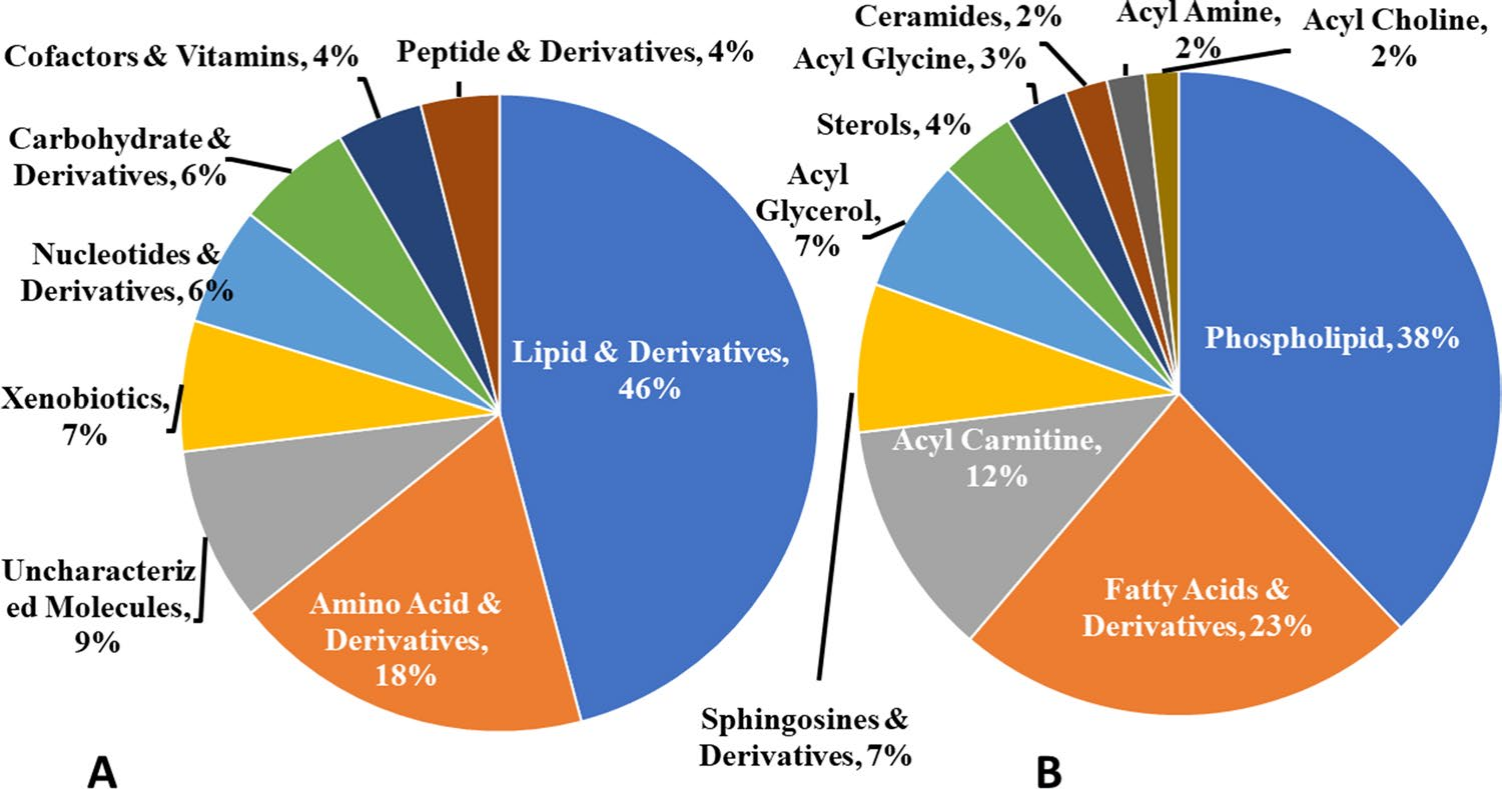
Pie chart illustrating the distribution of biochemicals detected in metabolomic profiling of kidney tissues. (**A**) Provides the percentage of all the metabolites detected, and (**B**) shows the subclassification of lipids and their derivatives.

We conducted a comprehensive metabolomic analysis of kidney tissues from mice raised in standard and hypoxic conditions. Metabolomic profiling identified 1029 biochemicals, but the chemical identity of 91 remained undetermined (labeled "uncharacterized molecules"). The comprehensive data can be found in the Supplementary Information 1. Figure 2A summarizes all metabolites identified through the untargeted metabolomic profiling, with nearly half of the identified metabolites being lipids. These lipids are further categorized by class (a comprehensive list is available in Supplementary Information 2). Figure 2B summarizes the lipids with their category. Furthermore, Tables S3 and S4 in Supplementary Information 3 provide the individual counts of each class of metabolites.

The overall results showed that 46% of the identified biochemicals belonged to lipids and their derivatives. Amino acids and derivatives constituted 18% of the metabolites. 7% of the biochemicals were classified as xenobiotic, the biochemicals found in metabolic profiling but are not naturally produced. 6% each belonged to the category of carbohydrates & derivatives and nucleotide & derivatives, whereas 4% each were classified as cofactors & vitamins and peptides & derivatives. Among the lipids, phospholipids were found to be the most abundant (38%), followed by fatty acid & derivatives (23%). Other lipids identified in metabolic profiling included acyl carnitines (12%), sphingosines and derivatives (7%), acyl glycerol (7%), sterols (4%), and acyl glycine (3%). Acylcholines, acyl amines, and ceramides constituted 2% each of the identified lipids.

### Effect of hypoxia on kidney metabolites

**Figure 3:**
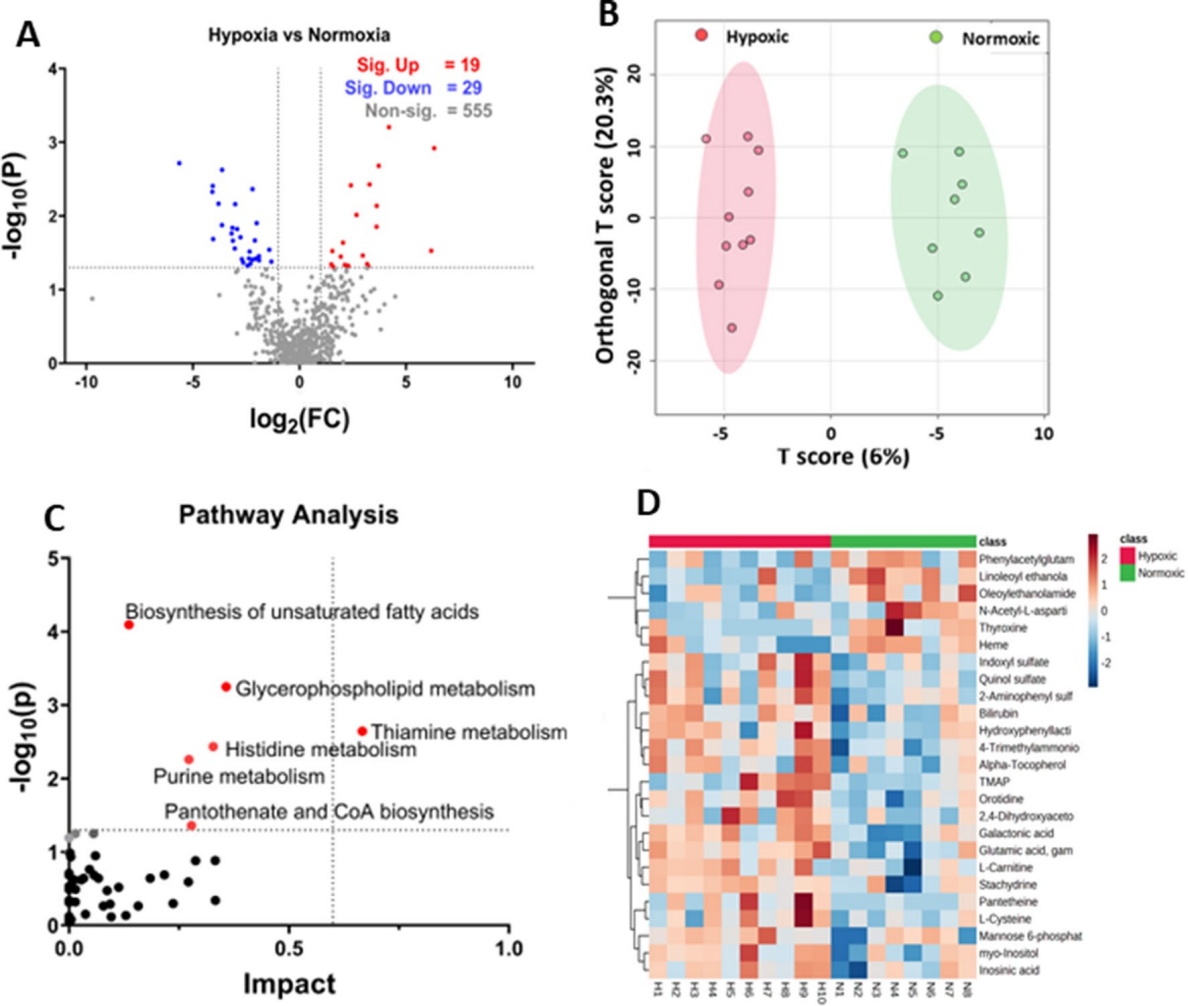
Analysis of hypoxic vs. normoxic groups. (**A**) Represents the volcano plot with 2FC and a p-value < 0.05, (**B**) represents the OPLS-DA score diagram between the hypoxic and normoxic groups. (**C**) The pathway analysis based on the VIP score from the OPLS-DA analysis. (**D**) Represents the heatmap of the top 25 significant features between the hypoxic and normoxic groups; log normalized intensity is presented in the red-blue colors, where red represents high and blue indicates low expressions.

Metabolites with known Human Metabolites Data Base (HMDB) identifiers were selected for further analysis. Using MetaboAnalyst 5.0, we conducted a univariate statistical analysis with t-tests to differentiate between hypoxic and normoxic groups, applying a two-fold (FC) change and a p-value cut-off of < 0.05. This analysis identified 19 biochemicals elevated under hypoxic conditions and 29 that were reduced (see Fig. 3A). Table 1 highlights significant metabolites, excluding lipids, whereas about half of the identified biochemicals, largely phospholipids, fatty acids, acyl glycerol, and sterols, are shown in Table S5 in Supplentary Information 3.

**Table 1:** Significantly altered non-lipid metabolites hypoxic (N = 10) vs. normoxic (N = 8) group using volcano plot analysis.

	Metabolite	log_2_(FC)	− Log_10_ (p)	Pathway/metabolite category
Significant increase	Pantetheine	6.1763	1.5268	Pantothenate and CoA biosynthesis
	4-Trimethylammoniobutanoic acid	3.6196	2.136	Carnitine synthesis
	l-Carnitine	3.6156	1.8515	Carnitine synthesis
	Galactonic acid	3.2893	2.4266	Galactitol and galactonate degradation
	Phenyllactic acid	3.1732	1.3458	Phenylalanine catabolism
	Orotidine	2.6719	2.014	Pyrimidine metabolism
	Glutamic acid gamma-methyl ester	2.4096	2.4153	Glutamic acid and derivatives
	Hydroxyphenyllactic acid	2.2837	1.3225	Phenylpropanoic acids
	Alpha-tocopherol	2.1247	1.3356	Active form of vitamin E
	myo-Inositol	2.0453	1.637	Inositol metabolism
	Inosinic acid	1.5923	1.3029	Purine metabolism
	Bilirubin	1.4957	1.3428	Porphyrin and heme metabolism
Significant decrease	Heme	− 1.4179	1.5407	Heme synthesis
	N-Acetyl-l-aspartic acid	− 2.444	1.3225	Aspartate metabolism
	Thyroxine	− 4.0523	1.6852	Thyroxine synthesis
	3 beta-Hydroxy-5-cholestenoate	− 5.6404	2.7161	Primary bile acid biosynthesis pathway

The Volcano plot analysis shows that pantetheine, involved in pantothenate and co-enzyme A biosynthesis, is the most significantly elevated (FC = 6.17, p-value = 0.0297) metabolite in the hypoxic group. Two metabolites involved in carnitine synthesis, 4-trimethylammoniobutanoic acid, and l-Carnitine, were increased in hypoxic groups, suggesting that carnitine synthesis was markedly increased in response to hypoxia. Other significantly upregulated metabolites included galactonic acid, phenyllactic acid, orotidine, glutamic acid gamma-methyl ester, hydroxyphenyllactic acid, alpha-tocopherol, myo-Inositol, inosinic acid, and bilirubin. The significantly downregulated metabolites included heme, N-Acetyl-l-aspartic acid, thyroxine, and 3-beta-Hydroxy-5-cholestenoate. Previous reports found similar results, discussed below.

We next performed the OPLS-DA analysis using MetaboAnalyst 5.0. The score diagram analysis shows a complete separation between the hypoxic and normoxic groups (see Fig. 3B).

#### Hypoxia-induced alterations in metabolites and metabolic pathways

The significant biochemical from OPLS-DA analysis were selected based on the variable importance in projection (VIP) > 1, as reported earlier by Hasegawa et al.^32^. Table S6 in the Supplementary Information 3 provides the list of these significant biochemicals. These significant biochemicals were used for Pathway Analysis from MetaboAnalyst 5.0., using a *Mus musculus* library containing 82 pathways. Table 2 shows the pathways that have a corresponding p-value < 0.05. The complete list of pathways is provided in Table S7 in Supplementary Information 3. Match status in Table 2 reflects hits/total, where total means the total number of compounds in the pathway, and the hits are the actually matched compounds from the list. The p-value reflects the raw p-value; Holm p is the adjusted p-value calculated from the Holm-Bonferroni method; the FDR means the False Discovery Rate; and the impact is the pathway impact value calculated from pathway analysis.

**Table 2:** Pathway analysis for hypoxic vs. normoxic group (N = 10 for hypoxic group and N = 8 for normoxic group).

Pathway name	Match status	p-value	− log10(p)	Holm p	FDR	Impact
Biosynthesis of unsaturated fatty acids	8/36	8.057E-5	4.0938	0.0067678	0.0067678	0.13636
Glycerophospholipid metabolism	7/36	5.6537E-4	3.2477	0.046926	0.023746	0.35739
Thiamine metabolism	3/7	0.0022688	2.6442	0.18604	0.063527	0.66667
Histidine metabolism	4/16	0.0036754	2.4347	0.2977	0.077183	0.32786
Purine metabolism	8/66	0.0055263	2.2576	0.4421	0.092842	0.27239
Pantothenate and CoA biosynthesis	3/19	0.043605	1.3605	1.0	0.59181	0.27857

The Pathway Analysis revealed that hypoxia induced the significant upregulation (p < 0.05) of several pathways (Fig. 3C), including biosynthesis of unsaturated fatty acids, glycerophospholipid, thiamine, histidine, and purine metabolism. Also, the pantothenate and CoA biosynthesis appeared upregulated in the pathway analysis. Considering FDR < 0.05, only two pathways appeared significantly upregulated due to hypoxia: biosynthesis of unsaturated fatty acids and glycerophospholipid metabolism. Previous reports on hypoxia-related studies revealed a similar pattern in metabolic pathway alterations, as discussed below.

Furthermore, a hierarchical clustering heat map was generated through MetaboAnalyst 5.0 that indicated visual clustering of hypoxic vs. normoxic groups (Fig. 3D). The rows in the heat map showed significantly altered metabolites, and the columns in the heat map represent biological replicates. The blue color represents the significantly downregulated metabolites, and the red color indicates the significantly upregulated metabolites; the intensity of the color corresponds to the log-normalized intensity of the metabolites, as shown in the bar in the top right corner of the heat map. A closer look at the heat map indicated a similar pattern observed in the volcano plot analysis. For instance, the visual inspection of the heat map showed that inosinic acid, myo-inositol, l-carnitine, and pantetheine are significantly upregulated in the hypoxic group, as shown by red color boxes in the lower left corner of the heat map. Also, N-acetyl-l-aspartic acid, thyroxine, and heme appear downregulated in the hypoxic group, as indicated by the light blue colors in the heat map.

### Sex-based metabolic profiling of hypoxia

We examined the data based on sex as well to see if hypoxia has different effects on male and female mice. The male group consisted of ten animals, of which five were raised in normoxic conditions, and five were exposed to hypoxic chambers. The Female group consisted of eight animals, of which 3 female mice were raised in normoxic conditions, and five were subjected to severe hypoxia.

#### Metabolic profiling of hypoxia in male mice

**Figure 4:**
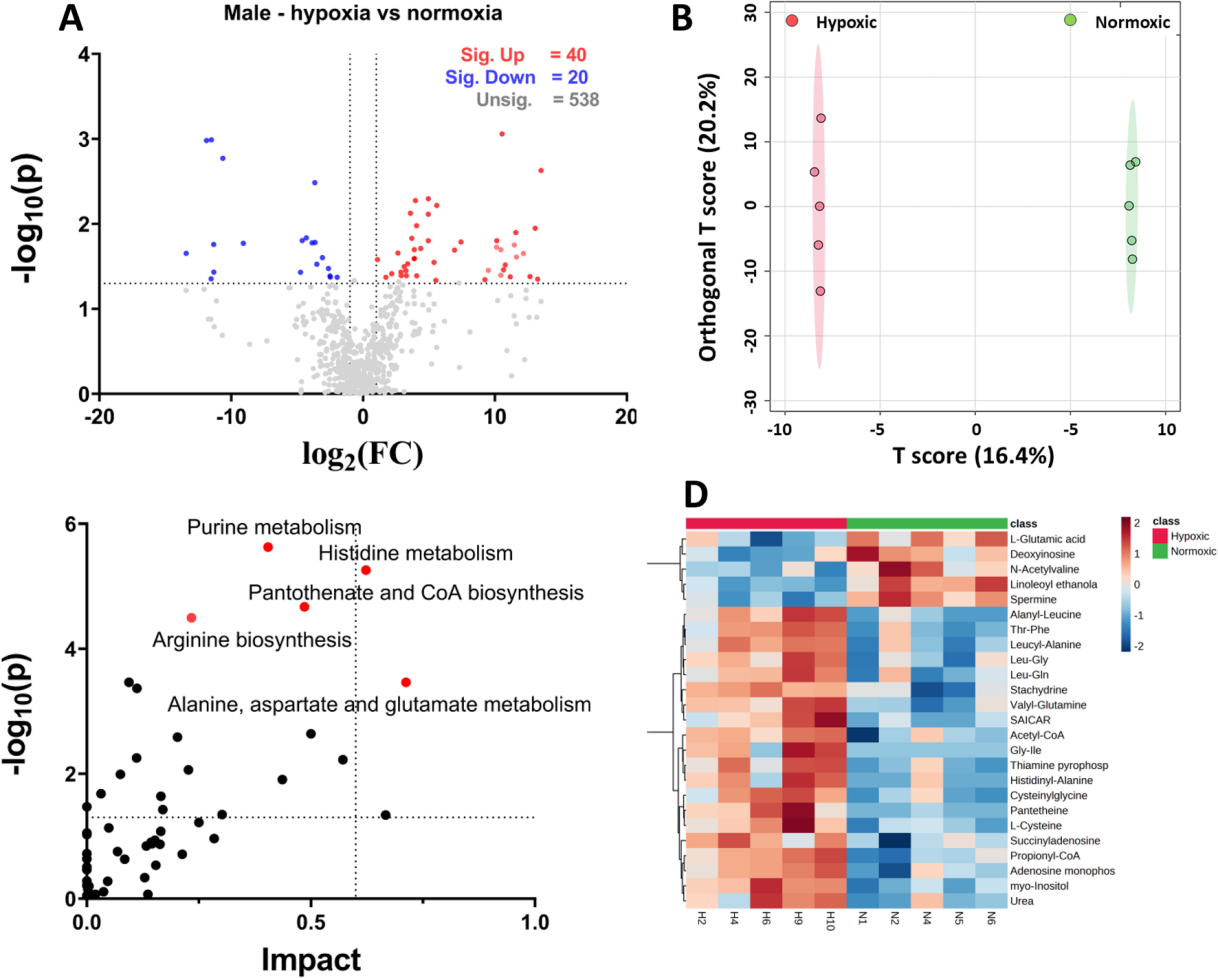
Analysis of hypoxic vs. normoxic groups in male mice. (**A**) Represents the volcano plot with 2FC and a p-value < 0.05, (**B**) represents the Orthogonal Partial Least Square Analysis between the hypoxic male and normoxic male group. (**C**) The pathway analysis. (**D**) Represents the heatmap of the top 25 significant features between the male hypoxic and male normoxic groups; log normalized intensity is presented in the red-blue colors, where red represents high, and blue indicates low expressions.

Volcano plot analysis with two-fold change and a cut-off p-value < 0.05 indicates 66 altered metabolites, of which 40 were significantly elevated in the hypoxic group, and 20 were significantly down (Fig. 4A; Table S8). As seen with the sex-independent hypoxic vs. normoxic group (Table S5 in Supplementary Information 3), many metabolites belonged to the lipid category (phospholipids, acylcarnitines, and fatty acids). Non-lipid elevated metabolites in the male hypoxic group, which were also seen elevated in the sex-independent hypoxic group, included pantetheine, 4-trimethylammoniobutanoic acid, galactonic acid, orotidine, glutamic acid gamma-methyl ester, alpha-tocopherol, and myo-inositol (Table 3). However, when observing downregulated metabolites in the male hypoxic group, only one metabolite appeared similar in the sex-independent hypoxic group, i.e., 3-beta-hydroxy-5-cholestenoate. The variation in results in the male group (10 mice) vs. the sex-independent group (18 mice) could be due to the fewer animals in this analysis.

**Table 3:** Significantly altered non-lipid biochemicals from Volcano plot with twofold change and p-value < 0.05 in male mice (N = 5 for each hypoxic and normoxic group.

	Biochemicals	log2 (FC)	− Log10 (P)	Pathway/metabolite category
Significant increase	Pantetheine	13.497	2.629	Pantothenate and CoA biosynthesis
	Sorbitol	13.232	1.3491	Fructose and mannose degradation
	SAICAR	13.051	1.9493	Purine metabolism
	Galactonic acid	12.16	1.6526	Galactitol and galactonate degradation
	l-Homoserine	11.466	1.7508	Methionine metabolism
	myo-Inositol	10.538	3.0587	Inositol metabolism
	Betaine	10.448	1.3958	Betaine metabolism
	Glutamic acid, gamma-methyl ester	9.4995	1.4527	Glutamic acid and derivatives
	l-Arabitol	9.2328	1.344	Sugar alcohol
	Urea	7.4236	1.7868	Urea cycle
	l-Cysteine	5.5896	2.2174	Methionine metabolism
	Adenosine monophosphate	4.944	2.1141	Purine metabolism
	Succinyladenosine	4.941	1.8023	Purine nucleoside
	4-Trimethylammoniobutanoic acid	4.3663	1.7112	Carnitine synthesis
	Acetyl-CoA	4.0512	1.9797	Fatty acid metabolism
	Inosinic acid	3.8745	1.6973	Purine metabolism
	Thiamine pyrophosphate	3.5825	2.1263	Thiamine metabolism
	Orotidine	3.3748	1.5296	Pyrimidine metabolism
	Uridine 5ʹ-monophosphate	2.8874	1.3929	Pyrimidine metabolism
	Guanosine monophosphate	2.8655	1.4323	Purine metabolism
	Alpha-Tocopherol	2.6389	1.6576	Active form of vitamin E
	AICAR	2.1741	1.4138	Purine metabolism
	N-Methyl-proline	1.7265	1.3718	Proline and derivatives
	l-Cysteinylglycine disulfide	1.0897	1.5796	Dipeptide
Significant decrease	Pyroglutamic acid	− 1.9716	1.3723	Glutathione metabolism
	Xylitol	− 2.4925	1.3737	Sugar alcohol
	l-Glutamic acid	− 3.641	1.7814	Glutamate metabolism
	3 beta-Hydroxy-5-cholestenoate	− 4.3007	1.8348	Primary bile acid biosynthesis pathway
	Cysteine-S-sulfate	− 4.7517	1.4311	Cysteine biosynthesis
	Deoxyinosine	− 9.085	1.7714	Purine metabolism
	Spermine	− 11.513	2.989	Arginine and proline metabolism
	Phosphoenolpyruvic acid	− 13.415	1.6534	Glycolysis

*SICAR* succinylaminoimidazolecarboxamide ribotide, *AICAR* 5-aminoimidazole-4-carboxamide ribonucleoside.

OPLS-DA showed a visible complete separation of features in the male hypoxic and normoxic groups that appeared tighter than sex-independent hypoxic and normoxic groups (Fig. 4B). The metabolites with the VIP score > 1 from OPLS_DA were selected for pathway analysis (Table S9). The Pathway Analysis revealed that purine metabolism, histidine metabolism, pantothenate and CoA biosynthesis, arginine biosynthesis, and alanine aspartate and glutamate metabolism were significantly upregulated (Fig. 4C). The upregulation of purine metabolism and pantothenate and CoA biosynthesis appeared similar to the sex-independent hypoxic vs. normoxic group. Table S10 in the Supplementary Information 3 provides the complete list of upregulated pathways.

Hierarchical clustering of metabolites was observed in the heat map (Fig. 4D), which showed better clustering of significantly altered metabolites in the male hypoxic vs. normoxic group. The pattern observed in the heat map seemed to be consistent with the volcano plot analysis.

#### Metabolic profiling of hypoxia in female mice

**Figure 5:**
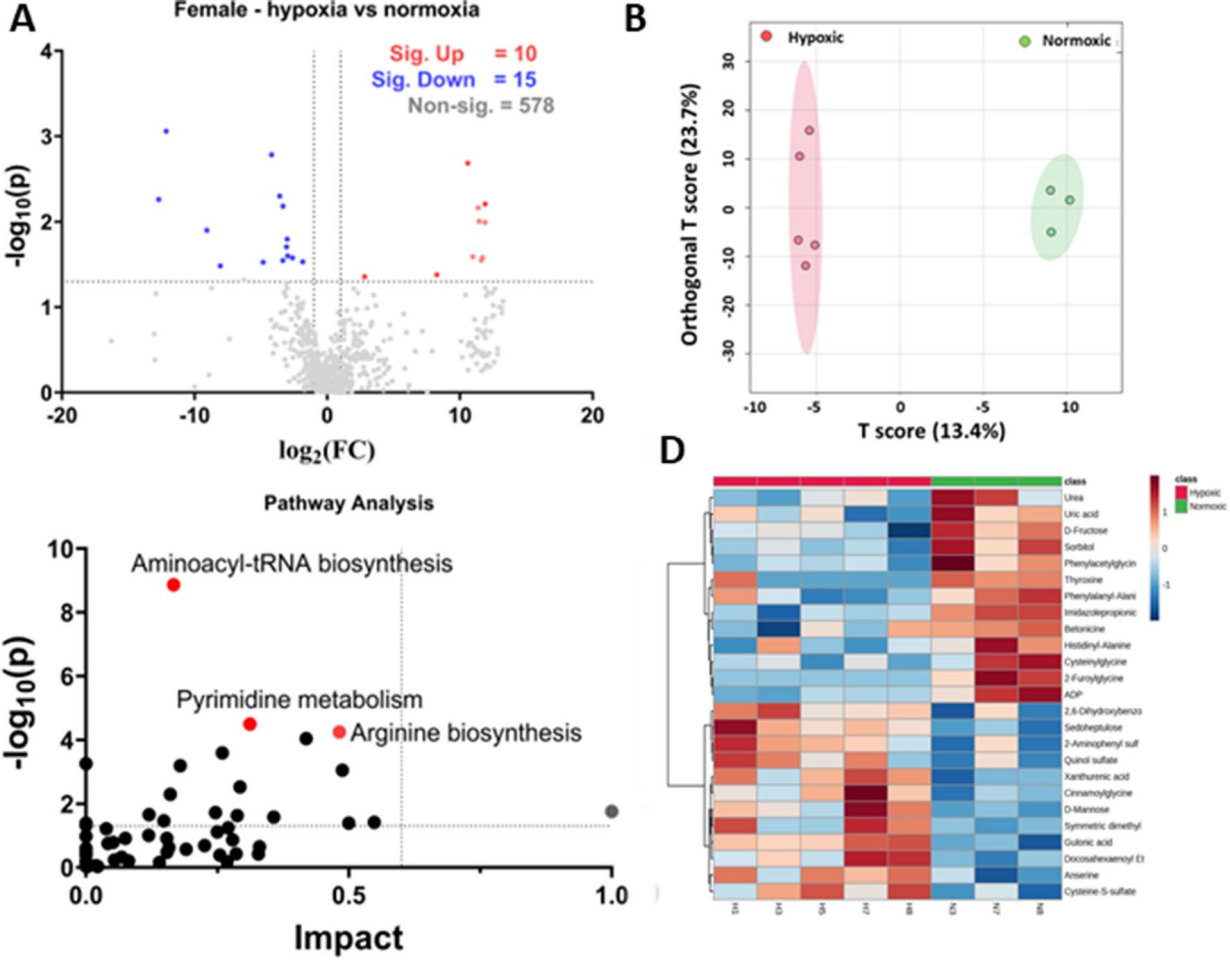
Analysis of hypoxic vs. normoxic in female mice. (**A**) Represents the volcano plot with 2FC and a p-value < 0.05, (**B**) Represents the OPLS-DA between the hypoxic and normoxic groups. (**C**) The pathway analysis based on VIP score < 1 from OPLS-DA analysis. (**D**) Represents the heatmap of the top 25 significant features between the hypoxic and normoxic groups; log normalized intensity is presented in the red-blue colors, where red represents high and blue indicates low expressions.

Volcano plot analysis with two-fold change and a p-value cut-off < 0.05 showed 25 altered metabolites, of which 10 are significantly upregulated and 15 are significantly down (Fig. 5A; Table S11). The observed altered metabolites were not similar to the sex-independent group or male group, which could be due to the lesser number of animals in the female hypoxic group (N = 8) vs. male hypoxic group (N = 10) and sex-independent hypoxic group (N = 18). Also, the number of animals in the female mice group was unequal, which could contribute to different results.

OPLS-DA analysis showed a distinctive separation of components between hypoxic and normoxic groups (Fig. 5B). The metabolites with VIP score > 1 are listed in Table S12 in the Supplementary Information 3. The pathway analysis showed that aminoacyl tRNA biosynthesis, pyrimidine metabolism, and arginine biosynthesis were significantly upregulated. (Fig. 5C). The upregulated pathways appeared different in the female group in comparison to the sex-independent group (Table S13). However, arginine biosynthesis appeared significantly upregulated in both males and females.

The heat map from the female group showed a distinctive pattern that appeared to be consistent with the volcano plot analysis, as represented by red and blue colors (Fig. 5D).

#### Covariate analysis of hypoxia according to sex

**Figure 6:**
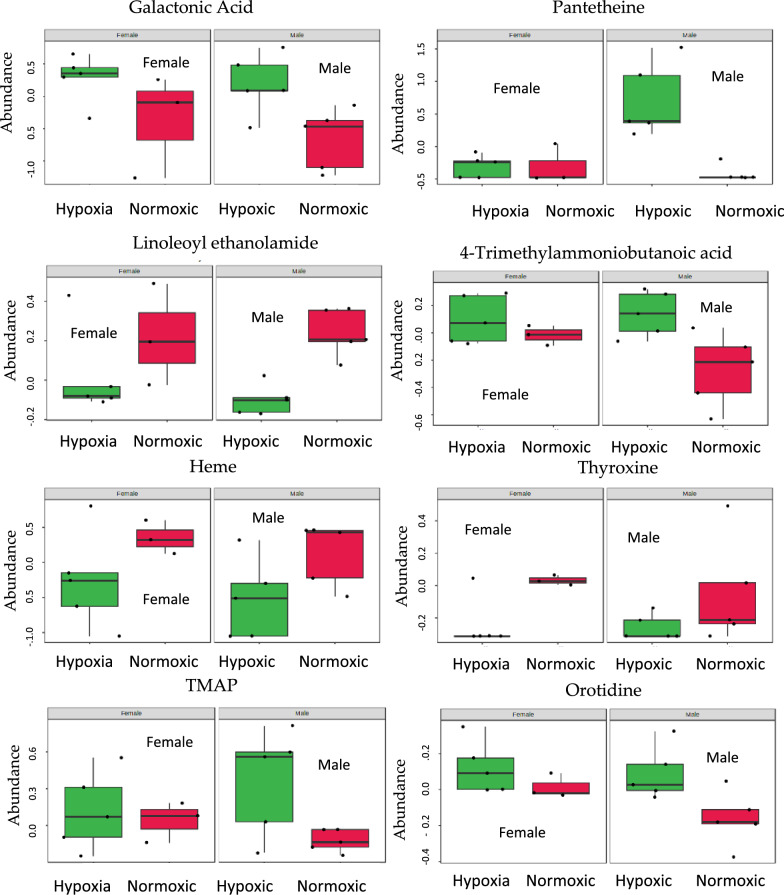
Box plots showing the effect of gender on condition (Hypoxic vs. Normoxic), using two-way ANOVA.

We used multivariate features from MetaboAnalyst 5.0 to examine the sex-based effect of hypoxia. Table S14 shows the list of metabolites that appear to be significant with a p-value of < 0.05. The results indicated alterations in 16 metabolites. Negative log_2_ fold change (FC) values indicated significantly elevated metabolites in hypoxic conditions in male and female kidneys, whereas positive log_2_ FC meant that the metabolites were significantly downregulated in both sexes. According to the co-variate analysis, galactonic acid, pantetheine, 4-trimethylammoniobutanoic acid, quinol sulfate, l-cysteine, orotidine, stachydrine, 2,4-dihydroxyacetophenone 5-sulfate, uridine diphosphate glucose (UDP), TMAP (N, N, N-Trimethyl-l-alanyl-l-proline betaine), indoxyl sulfate, and inosinic acid appeared to be elevated in both male and female kidneys in hypoxic condition. In contrast, linoleoyl ethanolamide, heme, oleoylethanolamide, and thyroxine appeared significantly decreased in male and female hypoxic kidneys (Fig. 6).

We generated the heatmap to visualize the effect of hypoxia on sex (Fig. S2). The heatmap does not show a clear clustering of metabolites according to sex. Although results from the male group, female group, and the co-variate analysis show sex-based differences in metabolites, it is difficult to deduce sex-specific metabolic alteration, possibly due to the lesser number of animals when classified according to sex.

### Biomarker discovery

**Figure 7:**
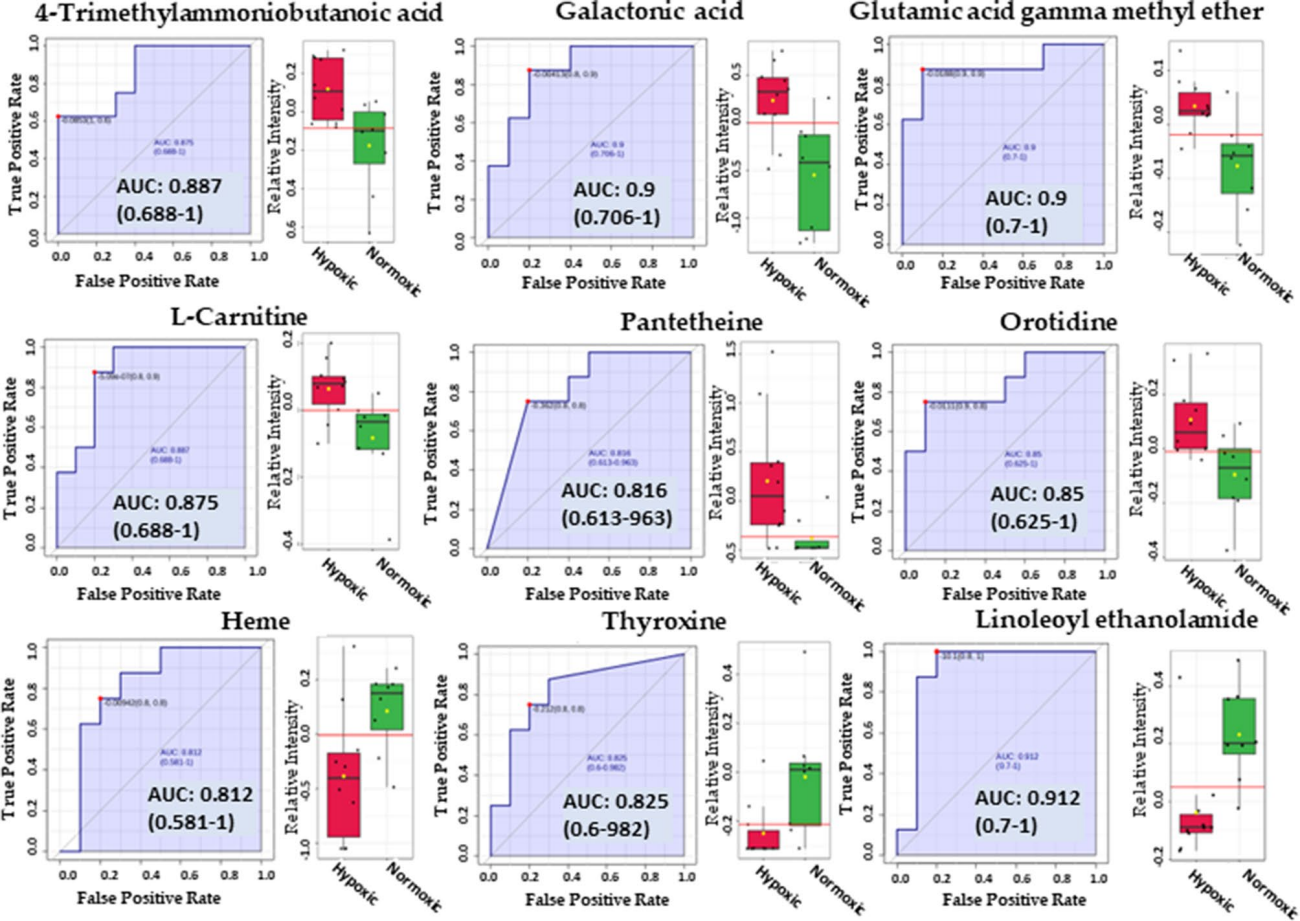
Biomarkers identified using the biomarker discovery feature of MetaboAnalyst 5.0.

We performed classical univariate ROC curve analysis to calculate AUC at 95% confidence intervals using MetaboAnalyst 5.0. One of the aims of the current study was to identify the biomarkers for hypoxia with high sensitivity (true-positive rate) and specificity (true-negative rate). As mentioned above, an AUC > 0.8 is considered a good predictor of a biomarker^26^; therefore, we selected the metabolites with an AUC > 0.8 as potential hypoxic biomarkers. Table S15 describes the biomarkers identified using the normalized data with their corresponding p-values < 0.05 from the t-tests and AUC > 0.8. Figure 7 shows the identified biomarkers with their corresponding box plots and ROC curves. The results appeared similar to those obtained from the volcano plot analysis from the sex-independent hypoxic vs. normoxic group (Table 1). It can be inferred from the results that 4-trimethylammoniobutanoic acid (p-value = 0.00731, AUC = 0.8625), galactonic acid (p-value = 0.00374, AUC = 0.875), glutamic acid gamma-methyl ester (p-value = 0.00384, AUC = 0.8875), pantetheine(p-value = 0.0297, AUC = 0.8125), l-carnitine (p-value = 0.0140, AUC = 0.875), and orotidine (p-value = 0.0096834, AUC = 0.8375) can serve as potential biomarkers for hypoxia as they appeared significantly elevated in hypoxic group compared to normoxic group (Fig. 7). However, linoleoyl ethanolamide (p-value = 0.00406, AUC = 0.9), heme (p-value = 0.028, AUC = 0.8125), and thyroxine (p-value = 0.020, AUC = 0.81875) appeared significantly decreased in the hypoxic group in comparison to the normoxic group.

## Discussion

We utilized the histological approach to understand the effect of hypoxia on kidney tissues. The Masson's Trichome Staining revealed that hypoxia caused significant fibrosis in both the cortex and medulla of the kidneys, consistent in male and female kidneys (Fig. 1). The results reported in this study align with the previous findings^4–6^. Furthermore, hypoxia causes limited mitochondrial metabolic water production, Krebs-Szent-Györgyi cycle molecular crowding and potential deuterium accumulation that have implications in oncogenic progression and fibrosis^35,36^ as observed in our study (Fig. 1). The untargeted metabolomic profiling identified 1029 biochemicals, with nearly half of the identified biochemicals belonging to the lipid category (Tables S3 and S4). The abundance of lipid metabolites was due to the latest metabolomic tools employing polar and non-polar extraction techniques with advanced chromatography^37^. The increased prevalence of lipids in the identified metabolites can also be attributed to the kidney tissue samples, as tissues contain more lipids than urine, plasma, or serum samples^38^.

Volcano plot analysis between sex-independent hypoxic and normoxic groups, applying a two-fold change and a p-value < 0.05, indicated 19 elevated biochemicals under hypoxic conditions, and 29 biochemicals were found to be significantly reduced. Many significantly altered metabolites were lipids consisting of phospholipids, fatty acids and derivates, acylcarnitines, acyl glycerol, and sterols (Table S4). Among the non-lipid metabolites, pantetheine, an activated form of vitamin B5 necessary for the synthesis of CoA, was found to be significantly elevated (FC = 6.1763, and − log_10_(p) = 1.5268) in the sex-independent hypoxic group. CoA is crucial for intracellular fat transport and energy metabolism^39^. Pantetheine also appeared to be significantly upregulated in male hypoxic kidneys (FC = 13.497, and − log_10_(p) = 2.629) (Table 3), but it was not observed elevated in the female hypoxic group, possibly due to a lesser number of animals in this group. Furthermore, Two-way ANOVA and Biomarker discovery analysis revealed pantetheine as a significantly elevated metabolite (p-value = 0.0297, AUC = 0.8125) (Figs. 6 and 7). These results align with the previous studies on pantetheine, suggesting that its levels are remarkably elevated in response to stress^40^, and pantetheine' administration restores CoA levels and improves mitochondrial function^41^.

In the sex-independent hypoxic group, 4-Trimethylammoniobutanoic acid and L-carnitine, both from the carnitine synthesis pathway, exhibited significant elevation, implying enhanced carnitine synthesis in response to hypoxic conditions. This is consistent with previous studies by Knabb et al. and Lou et al. that observed increased levels of acylcarnitines and carnitines during hypoxic conditions^42,43^. Furthermore, biomarker analysis revealed significant upregulation of 4-trimethylammoniobutanoic acid (p-value = 0.00731, AUC = 0.8625) and l-carnitine (p-value = 0.0140, AUC = 0.875). Notably, acylcarnitines rank third in abundance in our study (n = 56, Table S4). Historically identified over 70 years ago, acylcarnitines, which are believed to encompass over 1000 types in mammals, facilitate the transport of acyl groups for metabolism within the mitochondria^44,45^. Additionally, Glutamic acid gamma-methyl ester's elevation in the hypoxic group is consistent with Yang et al.'s suggestion of its potential as a biomarker for conditions associated with hypoxia, such as retinopathy of prematurity^46^.

**Figure 8:**
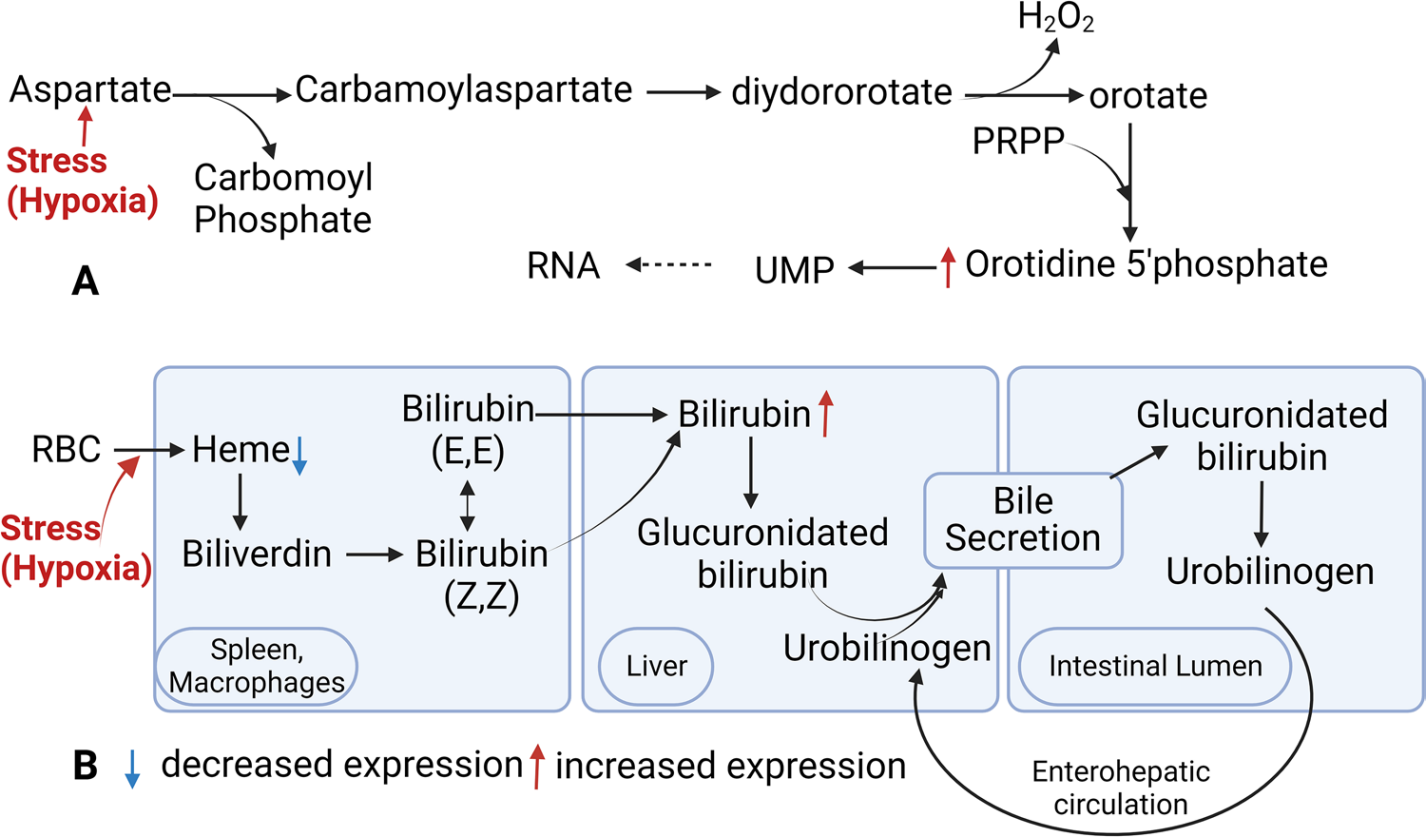
Schematic illustration of pathways. (**A**) Represents the pathway involving orotidine. (**B**) Represents the pathway involving heme and bilirubin.

Galactonic acid, a breakdown product of galactose, was notably elevated, consistent with Xing et al.'s findings of increased levels in chronic hypoxia, suggesting its potential as a hypoxia biomarker^47^. Phenyllactic acid (2-hydroxy-3-phenyl propionic acid; PLA), derived from phenylalanine catabolism^48^, was also elevated in the hypoxic group, aligning with Bakkeren et al.'s observations in newborns with respiratory distress^49^. Orotidine is a nucleoside formed by attaching orotic acid to a ribose ring via a beta-N1-glycosidic bond and has been associated with poor kidney function^50^. Our study reported significant orotidine upregulation in the hypoxic kidneys, which is consistent with the report by Shah et al., who proposed orotidine as a novel biomarker for cardiovascular disease risk prediction in type 2 diabetes through a metabolomic study^50^. It is presumed that stress (hypoxia) causes the upregulation of aspartate catabolism, leading to the elevation of orotidine (Fig. 8A). Furthermore, it is reported that glutamine-derived aspartate plays a crucial role in hypoxic conditions or environments causing electron transport chain (ETC) impairment^51^.

Heme, which breaks down via the oxygen-requiring heme oxygenase, was reduced in hypoxic conditions (Fig. 8B), possibly due to increased heme oxygenase induction^52,53^. Increased bilirubin levels observed in our study also suggest upregulation of heme catabolism^53^. N-acetyl-l-aspartic acid is significantly downregulated in the hypoxic group, consistent with the study of hypoxia on cerebral metabolites by Rosenberg et al.^54^ and Marcucci et al.^55^. Thyroxine is an important hormone reported to decrease significantly in high altitudes^56^, possibly due to the inactivation of local thyroid hormones by hypoxia Inducible Factor^57^.

The pathway analysis revealed that the biosynthesis of unsaturated fatty acids was significantly upregulated, which complies with the findings by Kamphorst et al., whereby they proposed that hypoxic cells promote the synthesis of unsaturated fatty acids by the breakdown of phospholipids^58^. The study also explained the upregulation of glycerophospholipid metabolism^58^, which may be due to the breakdown of phospholipids to provide free fatty acids for uptake by mitochondria through acyl-carnitines. Analyses of urinary metabolites in response to hypoxia by Lou et al. reported upregulation of histidine and purine metabolism^43^; a similar pattern was observed in our study. Lou et al. proposed a link between the upregulation of these pathways and the induction of Hypoxia-Induced Factor-1(HIF-1)^43^. Fan et al. recently reported the enrichment of biosynthesis of unsaturated fatty acids and histidine metabolism during their study on exosome metabolites hypoxic preconditioning participants^59^.

Audano et al. comprehensively reviewed the sex-related metabolomic and lipidomic profiles and concluded that sufficient experimental evidence pointed to sex as one of the most significant biological variables influencing metabolomic and lipidomic profiles^38^. Our study indicated a wide variation in metabolite distribution in male and female kidneys. Considering p-value < 0.05 as a measure of significance in two-way ANOVA showed sex-independent elevation of some metabolites (Fig. 6); however, using FDR < 0.05 did not show any common metabolite between the genders that were significantly altered, this observation could be due to a small number of animals in male and female groups. Further studies are required to discover sex-specific hypoxia biomarkers.

## Conclusion

High Altitude Renal Syndrome is characterized by several cardiovascular and renal ailments. Chronic hypoxia is believed to affect kidney function and cause renal fibrosis. In this study, we examined the kidney tissues of mice exposed to chronic hypoxia by placing them in specifically designed hypoxia chambers. The results of Trichome staining reveal significant fibrosis when compared with the kidney tissues obtained from mice raised under normoxic conditions. Comprehensive metabolic profiling of kidney tissues showed several metabolites that were significantly upregulated. Notable among these metabolites are 4-Trimethylammoniobutanoic acid, l-carnitine, pantetheine, galactonic acid, orotidine, alpha-tocopherol, myo-inositol, inosinic acid, and bilirubin. The biomarker discovery feature of MetaboAnalyst 5.0 also revealed the discovery of these metabolites, and it can be reasonably argued that these metabolites can serve as biomarkers for hypoxia.

Furthermore, several research reports investigating hypoxia in different disease conditions showed similar patterns as our study observed, further strengthening the argument. Pathway Analysis based on a comprehensive and statistically robust OPLS-DA indicates that histidine and purine metabolism are significantly upregulated when the mice are exposed to severe hypoxia. We also investigated the effects of hypoxia on male and female mice; the results reveal a wide variation in metabolite distribution. Considering p-value < 1 in two-way ANOVA showed significantly altered metabolites independent of sex, but using FDR < 0.05 did not reveal sex-independent metabolites that could be associated with hypoxic conditions. This observation could be due to a small number of animals in male and female groups. Further studies are needed to determine whether hypoxia affects males and females differently. Finally, the present study provides evidence that robust metabolomic studies with the simultaneous advancements in UP-HPLC, bioinformatics tools, databases, and highly sensitive and specific identification of biochemicals can lead to the discovery of novel biomarkers for different disease conditions that will improve real-time diagnosis and management of diseases.

### Supplementary Information

Supplementary Information 1.

Supplementary Information 2.

Supplementary Information 3.

## Data Availability

All data generated or analyzed during this study are included in this published article and its supplementary information files. Appendix A is uploaded as an Excel file containing the raw data of biochemicals; the first tab of the Excel sheets provides the explanations for the rest of the tabs in this excel sheet named “Data.” Appendix B is uploaded as an Excel file containing the lipids detected in the current study and their classification; the sheet is named “Lipids”.

## References

[1] Luks AM, Johnson RJ, Swenson ER (2008). Chronic kidney disease at high altitude. J. Am. Soc. Nephrol..

[2] Arestegui AH (2011). High altitude renal syndrome (HARS). J. Am. Soc. Nephrol..

[3] Wang S-Y, Gao J, Zhao J-H (2022). Effects of high altitude on renal physiology and kidney diseases. Front. Physiol..

[4] Fine L, Orphanides C, Norman J (1998). Progressive renal disease: The chronic hypoxia hypothesis. Kidney Int. Suppl..

[5] Fine LG, Norman JT (2008). Chronic hypoxia as a mechanism of progression of chronic kidney diseases: from hypothesis to novel therapeutics. Kidney Int..

[6] Shamloo K (2017). Chronic hypobaric hypoxia modulates primary cilia differently in adult and fetal ovine kidneys. Front. Physiol..

[7] Ow CP, Ngo JP, Ullah MM, Hilliard LM, Evans RG (2018). Renal hypoxia in kidney disease: Cause or consequence?. Acta Physiol..

[8] Wheaton, W. W. & Chandel, N. S. Hypoxia. 2. Hypoxia regulates cellular metabolism. *Am. J. Physiol. Cell Physiol.* **300**, C385–C393 (2011). 10.1152/ajpcell.00485.2010 PMC3063979 21123733

[9] Solaini, G., Baracca, A., Lenaz, G. & Sgarbi, G. Hypoxia and mitochondrial oxidative metabolism. *Biochim. Biophys. Acta (BBA)-Bioenergetics* **1797**, 1171–1177 (2010). 10.1016/j.bbabio.2010.02.011 20153717

[10] Chen P-S (2020). Pathophysiological implications of hypoxia in human diseases. J. Biomed. Sci..

[11] Beneduci A, Cuccurullo M, Pontoni G, Chidichimo G, Capasso G (2010). Perspectives of 1H-NMR-based urinary metabonomics in Fabry disease. J. Nephrol..

[12] Li, J., Ren, L. J., Sun, G.-N., Qu, L. & Huang, H. Comparative metabolomics analysis of docosahexaenoic acid fermentation processes by Schizochytrium sp. under different oxygen availability conditions. *Omics J. Integr. Biol.* **17**, 269–281 (2013). 10.1089/omi.2012.0088 23586678

[13] Liu X, Locasale JW (2017). Metabolomics: A primer. Trends Biochem. Sci..

[14] Jang C (2016). A branched-chain amino acid metabolite drives vascular fatty acid transport and causes insulin resistance. Nat. Med..

[15] Guma M, Tiziani S, Firestein GS (2016). Metabolomics in rheumatic diseases: Desperately seeking biomarkers. Nat. Rev. Rheumatol..

[16] Gaul DA (2015). Highly-accurate metabolomic detection of early-stage ovarian cancer. Sci. Rep..

[17] Reçber T, Nemutlu E, Beksac K, Aksoy S, Kır S (2020). Optimization and validation of a HILIC-LC-ESI-MS/MS method for the simultaneous analysis of targeted metabolites: Cross validation of untargeted metabolomic studies for early diagnosis of breast cancer. Microchem. J..

[18] Kim O (2020). In vivo modeling of metastatic human high-grade serous ovarian cancer in mice. PLoS Genet..

[19] Huang D, Gaul DA, Nan H, Kim J, Fernández FM (2019). Deep metabolomics of a high-grade serous ovarian cancer triple-knockout mouse model. J. Proteome Res..

[20] Arnegard ME, Whitten LA, Hunter C, Clayton JA (2020). Sex as a biological variable: A 5-year progress report and call to action. J. Women's Health.

[21] Albus, U. (SAGE Publications Sage UK: London, England, 2012).

[22] Percie du Sert N (2020). The ARRIVE guidelines 2.0: Updated guidelines for reporting animal research. J. Cereb. Blood Flow Metab..

[23] Amirrad F, Pala R, Shamloo K, Muntean BS, Nauli SM (2021). Arrhythmogenic hearts in PKD2 mutant mice are characterized by cardiac fibrosis, systolic, and diastolic dysfunctions. Front. Cardiovasc. Med..

[24] Evans AM (2014). High resolution mass spectrometry improves data quantity and quality as compared to unit mass resolution mass spectrometry in high-throughput profiling metabolomics. Metabolomics.

[25] Ford L (2020). Precision of a clinical metabolomics profiling platform for use in the identification of inborn errors of metabolism. J. Appl. Lab. Med..

[26] Xia J, Broadhurst DI, Wilson M, Wishart DS (2013). Translational biomarker discovery in clinical metabolomics: An introductory tutorial. Metabolomics.

[27] Boccard J, Rutledge DN (2013). A consensus orthogonal partial least squares discriminant analysis (OPLS-DA) strategy for multiblock Omics data fusion. Anal. Chim. Acta.

[28] Jonsson P (2005). Extraction, interpretation and validation of information for comparing samples in metabolic LC/MS data sets. Analyst.

[29] Pérez-Enciso M, Tenenhaus M (2003). Prediction of clinical outcome with microarray data: A partial least squares discriminant analysis (PLS-DA) approach. Hum. Genet..

[30] Bylesjö M (2006). OPLS discriminant analysis: Combining the strengths of PLS-DA and SIMCA classification. J. Chem. Soc..

[31] Trygg J, Wold S (2002). Orthogonal projections to latent structures (O-PLS). J. Chem. Soc..

[32] Hasegawa S (2020). The oral hypoxia-inducible factor prolyl hydroxylase inhibitor enarodustat counteracts alterations in renal energy metabolism in the early stages of diabetic kidney disease. Kidney Int..

[33] Pang Z (2022). Using MetaboAnalyst 5.0 for LC–HRMS spectra processing, multi-omics integration and covariate adjustment of global metabolomics data. Nat. Protocols.

[34] Lackner J, Hess V, Marx A, Hosseini-Ghaffari M, Sauerwein H (2022). Effects of dietary supplementation with histidine and β-alanine on blood plasma metabolome of broiler chickens at different ages. Plos One.

[35] Boros LG (2016). Submolecular regulation of cell transformation by deuterium depleting water exchange reactions in the tricarboxylic acid substrate cycle. Med. Hypotheses.

[36] Aprile S (2022). An unexpected deuterium-induced metabolic switch in doxophylline. ACS Med. Chem. Lett..

[37] Leuthold P (2017). Comprehensive metabolomic and lipidomic profiling of human kidney tissue: A platform comparison. J. Proteome Res..

[38] Audano M, Maldini M, De Fabiani E, Mitro N, Caruso D (2018). Gender-related metabolomics and lipidomics: From experimental animal models to clinical evidence. J. Proteom..

[39] Neess D, Bek S, Engelsby H, Gallego SF, Faergeman NJ (2015). Long-chain acyl-CoA esters in metabolism and signaling: Role of acyl-CoA binding proteins. Prog. Lipid Res..

[40] Naquet P, Pitari G, Dupre S, Galland F (2014). Role of the Vnn1 pantetheinase in tissue tolerance to stress. Biochem. Soc. Trans..

[41] Rana A (2010). Pantethine rescues a Drosophila model for pantothenate kinase-associated neurodegeneration. Proc. Natl. Acad. Sci..

[42] Knabb MT, Saffitz JE, Corr PB, Sobel BE (1986). The dependence of electrophysiological derangements on accumulation of endogenous long-chain acyl carnitine in hypoxic neonatal rat myocytes. Circ. Res..

[43] Lou B-S, Wu P-S, Liu Y, Wang J-S (2014). Effects of acute systematic hypoxia on human urinary metabolites using LC–MS-based metabolomics. High Altitude Med. Biol..

[44] Dambrova M (2022). Acylcarnitines: Nomenclature, biomarkers, therapeutic potential, drug targets, and clinical trials. Pharmacol. Rev..

[45] Fritz IB (1959). Action of carnitine on long chain fatty acid oxidation by liver. Am. J. Physiol. Legacy Content.

[46] Yang Y (2022). Comparative analysis reveals novel changes in plasma metabolites and metabolomic networks of infants with retinopathy of prematurity. Investig. Ophthalmol. Vis. Sci..

[47] Xing J (2018). Hypoxia induces senescence of bone marrow mesenchymal stem cells via altered gut microbiota. Nat. Commun..

[48] Jung S, Hwang H, Lee J-H (2019). Effect of lactic acid bacteria on phenyllactic acid production in kimchi. Food Control.

[49] Bakkeren J, Sengers R, Trijbels J, Engels PT (1977). Organic aciduria in hypoxic premature newborns simulating an inborn error of metabolism. Eur. J. Pediatr..

[50] Shah HS (2022). Serum orotidine: A novel biomarker of increased CVD risk in type 2 diabetes discovered through metabolomics studies. Diabetes Care.

[51] Sullivan LB (2015). Supporting aspartate biosynthesis is an essential function of respiration in proliferating cells. Cell.

[52] Maines MD, Gibbs PE (2005). 30 some years of heme oxygenase: From a “molecular wrecking ball” to a “mesmerizing” trigger of cellular events. Biochem. Biophys. Res. Commun..

[53] Neubauer JA, Sunderram J (2012). Heme oxygenase-1 and chronic hypoxia. Respir. Physiol. Neurobiol..

[54] Rosenberg GA, White J, Gasparovic C, Crisostomo EA, Griffey RH (1991). Effect of hypoxia on cerebral metabolites measured by proton nuclear magnetic resonance spectroscopy in rats. Stroke.

[55] Marcucci F, Colombo L, De Ponte G, Mussini E (1984). Decrease in N-acetyl-l-aspartic acid in brain of myodystrophic mice. J. Neurochem..

[56] Connors JM, Martin LG (1982). Altitude-induced changes in plasma thyroxine, 3, 5, 3'-triiodothyronine, and thyrotropin in rats. J. Appl. Physiol..

[57] Simonides WS (2008). Hypoxia-inducible factor induces local thyroid hormone inactivation during hypoxic-ischemic disease in rats. J. Clin. Invest..

[58] Kamphorst JJ (2013). Hypoxic and Ras-transformed cells support growth by scavenging unsaturated fatty acids from lysophospholipids. Proc. Natl. Acad. Sci..

[59] Fan F (2023). Metabolomic and proteomic identification of serum exosome for hypoxic preconditioning participants. Oxid. Med. Cell. Long..

