# Supplementary material for: Untargeted metabolomics analysis on kidney tissues from mice reveals potential hypoxia biomarkers
Source: Sci Rep. 2023 Oct 16;13:17516. doi: 10.1038/s41598-023-44629-y (PMC10579359; doi:10.1038/s41598-023-44629-y)
Supplement: Supplementary file 3 — Supplementary Information 3. [file 41598_2023_44629_MOESM3_ESM.docx]

Supplementary Information

Untargeted Metabolomics Analysis on Kidney Tissues from mice reveals potential hypoxia biomarkers.

Muhammad Imran Sajid^#,1,2^, Francisco J. Nunez^#,1^, Farideh Amirrad^#,1^, Moom Rahman Roosan^1^, Tom Vojtko^3^, Scott McCulloch^3^, Amal Alachkar^4*^, and Surya M. Nauli^1,5*^

**
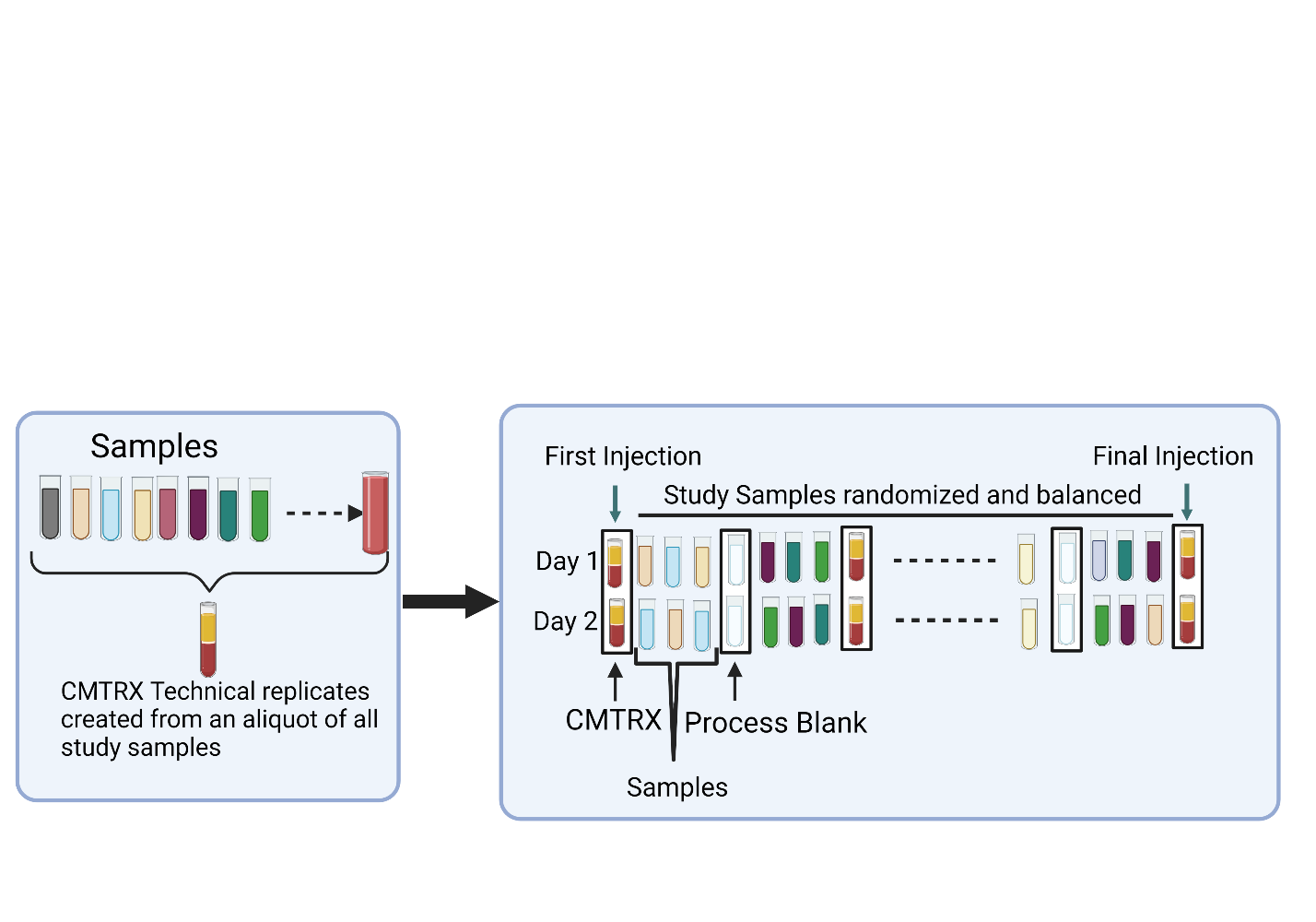
Figure S1**. Preparation of sample-specific technical replicates. A small aliquot of each sample (colored cylinders) is pooled to create a CMTRX technical replicate sample (multi-colored cylinder), then injected periodically throughout the platform run. Variability among consistently detected biochemicals can be used to estimate overall process and platform variability.

**
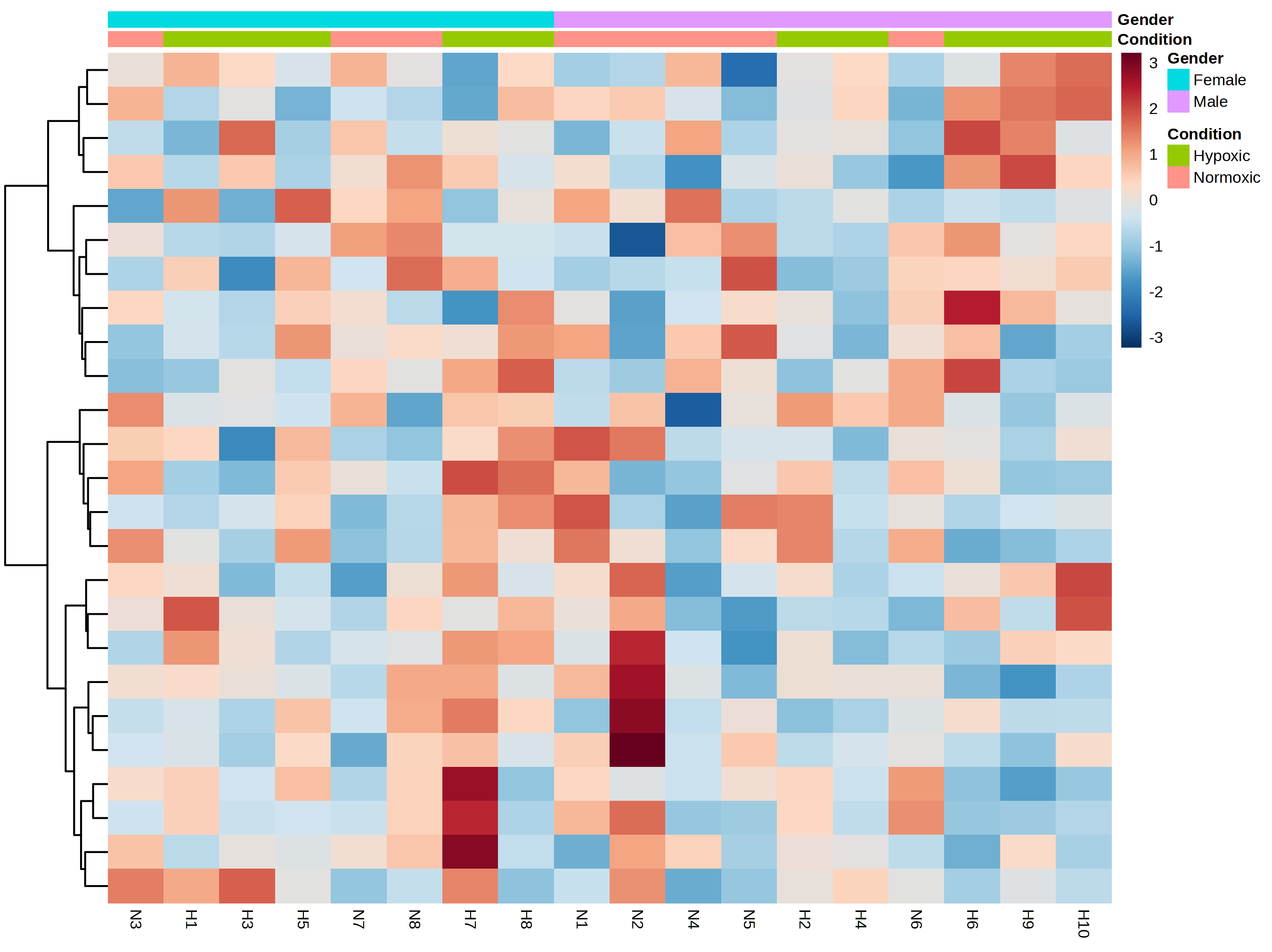
Figure S2** shows the heatmap from two-way ANOVA; the heatmap illustrates that gender does not appear to contribute towards the clustering of the metabolites.

**Table S1.** Description of Metabolon QC Samples

| **Type** | **Description** | **Purpose** |
| --- | --- | --- |
| MTRX | The large pool of plasma maintained by Metabolon has been characterized extensively. | Assure that all aspects of the Metabolon process are operating within specifications. |
| CMTRX | The pool was created by taking a small aliquot from every sample. | Assess the effect of a non-plasma matrix on the Metabolon process and distinguish biological variability from process variability. |
| PRCS | Aliquot of ultra-pure water | Process Blank was used to assess the contribution to compound signals from the process. |
| SOLV | Aliquot of solvents used in extraction. | Solvent Blank was used to segregate contamination sources in the extraction. |

**Table S2.** Metabolon QC Standards

| **Type** | **Description** | **Purpose** |
| --- | --- | --- |
| RS | Recovery Standard | Assess variability and verify the performance of extraction and instrumentation. |
| IS | Internal Standard | Assess the variability and performance of the instrument. |

**Table S3.** Distribution of detected biochemicals

| **Biochemicals** | **Count** |
| --- | --- |
| Lipid & Derivatives | 472 |
| Amino Acid & Derivatives | 189 |
| Uncharacterized Molecules | 91 |
| Xenobiotics | 68 |
| Nucleotides & Derivatives | 62 |
| Carbohydrate & Derivatives | 61 |
| Cofactors & Vitamins | 45 |
| Peptide & Derivatives | 41 |
| **Grand Total** | **1029** |

**Table S4.** Distribution of lipids and their derivatives

| **Lipids and their derivatives** | **Count** |
| --- | --- |
| Phospholipid | 179 |
| Fatty Acids & Derivatives | 110 |
| Acyl Carnitine | 56 |
| Sphingosines & Derivatives | 35 |
| Acyl Glycerol | 32 |
| Sterols | 18 |
| Acyl Glycine | 15 |
| Ceramides | 10 |
| Acyl Amine | 9 |
| Acyl Choline | 8 |
| **Grand Total** | **472** |

**Table S5** Significantly up and down biochemicals from Volcano plot with 2-Fold Change and p-value<0.05 for hypoxic vs. normoxic group (N=10 for hypoxic group; N=8 for normoxic group). Red fonts represent the upregulated metabolites, and the blue fonts represent the downregulated metabolites.

| Metabolite | log2(FC) | -Log10 (P) | Pathway/ Metabolite Category |
| --- | --- | --- | --- |
| PC(18:0/22:5(4Z,7Z,10Z,13Z,16Z)) | 6.3204 | 2.9201 | Phospholipid |
| Pantetheine | 6.1763 | 1.5268 | Pantothenate and CoA Biosynthesis |
| PC(18:2(9Z,12Z)/18:3(9Z,12Z,15Z)) | 4.1932 | 3.2048 | Phospholipid |
| PC(P-18:0/22:6(4Z,7Z,10Z,13Z,16Z,19Z)) | 3.7188 | 2.6803 | Phospholipid |
| 4-Trimethylammoniobutanoic acid | 3.6196 | 2.136 | Carnitine Synthesis |
| L-Carnitine | 3.6156 | 1.8515 | Carnitine Synthesis |
| Galactonic acid | 3.2893 | 2.4266 | Galactitol and Galactonate Degradation |
| SM(d18:2(4E,14Z)/23:0) | 3.2286 | 1.3141 | Sphingolipid |
| Phenyllactic acid | 3.1732 | 1.3458 | phenylalanine catabolism, |
| PC(18:2(9Z,12Z)/18:2(9Z,12Z)) | 2.9672 | 1.4605 | Phospholipid |
| Orotidine | 2.6719 | 2.014 | Pyrimidine Metabolism |
| Glutamic acid, gamma-methyl ester | 2.4096 | 2.4153 | Glutamic acid and derivatives |
| Hydroxyphenyllactic acid | 2.2837 | 1.3225 | Phenylpropanoic acids |
| Alpha-Tocopherol | 2.1247 | 1.3356 | Active form of Vitamin E |
| myo-Inositol | 2.0453 | 1.637 | Inositol Metabolism |
| PI(16:0/18:1(9Z)) | 1.9334 | 1.4469 | Phospholipid |
| Inosinic acid | 1.5923 | 1.3029 | Purine Metabolism |
| PC(15:0/18:2(9Z,12Z)) | 1.5385 | 1.5224 | Phospholipid |
| Bilirubin | 1.4957 | 1.3428 | Porphyrin and Heme Metabolism |
| 1-Oleoylglycerophosphoinositol | -1.3216 | 1.3777 | Phospholipid |
| Heme | -1.4179 | 1.5407 | Heme Synthesis |
| MG(0:0/22:6(4Z,7Z,10Z,13Z,16Z,19Z)/0:0) | -1.8962 | 1.3965 | Acyl Glycerol |
| LysoPS(16:0/0:0) | -1.8996 | 1.4516 | Lysophospholipid |
| 2-Stearoylglycerophosphoinositol | -2.01 | 1.9012 | Phospholipid |
| LysoPC(20:3(8Z,11Z,14Z)) | -2.0548 | 1.4175 | Lysophospholipid |
| LysoPI(18:0/0:0) | -2.095 | 1.6664 | Lysophospholipid |
| 2-Arachidonylglycerol | -2.1957 | 1.4084 | Acyl Glycerol |
| LysoPE(0:0/18:0) | -2.2065 | 2.3625 | Lysophospholipid |
| Palmitic acid | -2.297 | 1.353 | Fatty Acid |
| MG(16:0/0:0/0:0) | -2.3436 | 1.5141 | Acyl Glycerol |
| MG(20:4(5Z,8Z,11Z,14Z)/0:0/0:0) | -2.3762 | 1.4216 | Acyl Glycerol |
| N-Acetyl-L-aspartic acid | -2.444 | 1.3225 | Aspartate Metabolism |
| LysoPE(20:4(5Z,8Z,11Z,14Z)/0:0) | -2.6374 | 1.3663 | Lysophospholipid |
| Stearic acid | -2.7007 | 1.4131 | Fatty Acid |
| MG(22:6(4Z,7Z,10Z,13Z,16Z,19Z)/0:0/0:0) | -2.7704 | 1.7102 | Acyl Glycerol |
| LysoPC(22:6(4Z,7Z,10Z,13Z,16Z,19Z)) | -2.9266 | 1.8201 | Lysophospholipid |
| LysoPG(18:1(9Z)/0:0) | -3.0153 | 2.158 | Lysophospholipid |
| Eicosadienoic acid | -3.0341 | 1.5581 | Fatty Acid |
| 2-Palmitoylglycerophosphocholine | -3.126 | 1.6617 | Phospholipid |
| 8,11,14-Eicosatrienoic acid | -3.1467 | 1.837 | Fatty Acid |
| LysoPS(18:2(9Z,12Z)/0:0) | -3.1842 | 1.7612 | Lysophospholipid |
| Eicosapentaenoic acid | -3.6311 | 2.6245 | Fatty Acid |
| 1-Arachidonoylglycerophosphoinositol | -3.6384 | 1.874 | Phospholipid |
| Docosapentaenoic acid | -3.8017 | 2.1648 | Fatty Acid |
| Thyroxine | -4.0523 | 1.6852 | Thyroxine Synthesis |
| Docosahexaenoic acid | -4.0717 | 2.4063 | Fatty Acid |
| 6,9,12,15,18,21-Tetracosahexaenoic acid | -4.0873 | 2.3272 | Fatty Acid |
| 3 beta-Hydroxy-5-cholestenoate | -5.6404 | 2.7161 | primary bile acid biosynthesis pathway |

|  |
| --- |

**Table S6** Significant features from OPLS VIP score >1, used for pathway analysis hypoxic vs. normoxic group (N=10 for hypoxic group and N=8 for normoxic group).

| Significant Features | V1P score |
| --- | --- |
| PC(18:2(9Z,12Z)/18:3(9Z,12Z,15Z)) | 2.76071 |
| PC(18:0/22:5(4Z,7Z,10Z,13Z,16Z)) | 2.646255 |
| 3 beta-Hydroxy-5-cholestenoate | 2.59424 |
| PC(P-18:0/22:6(4Z,7Z,10Z,13Z,16Z,19Z)) | 2.575764 |
| Eicosapentaenoic acid | 2.565981 |
| LysoPE(0:0/18:0) | 2.489691 |
| Docosahexaenoic acid | 2.465427 |
| Linoleoyl ethanolamide | 2.457991 |
| Glutamic acid, gamma-methyl ester | 2.453881 |
| Galactonic acid | 2.452229 |
| 6,9,12,15,18,21-Tetracosahexaenoic acid | 2.450299 |
| LysoPG(18:1(9Z)/0:0) | 2.357982 |
| Docosapentaenoic acid | 2.350333 |
| 4-Trimethylammoniobutanoic acid | 2.322127 |
| Orotidine | 2.229164 |
| 2-Stearoylglycerophosphoinositol | 2.224292 |
| 8,11,14-Eicosatrienoic acid | 2.210602 |
| 1-Arachidonoylglycerophosphoinositol | 2.203416 |
| LysoPC(22:6(4Z,7Z,10Z,13Z,16Z,19Z)) | 2.169454 |
| Oleoylethanolamide | 2.162805 |
| LysoPS(18:2(9Z,12Z)/0:0) | 2.1441 |
| L-Carnitine | 2.139948 |
| 1-Palmitoylglycerophosphoinositol | 2.137458 |
| MG(22:6(4Z,7Z,10Z,13Z,16Z,19Z)/0:0/0:0) | 2.111906 |
| LysoPI(18:0/0:0) | 2.10427 |
| 2-Palmitoylglycerophosphocholine | 2.082083 |
| Thyroxine | 2.048247 |
| myo-Inositol | 2.028804 |
| Quinol sulfate | 2.025187 |
| Eicosadienoic acid | 2.003313 |
| Stachydrine | 1.970282 |
| MG(16:0/0:0/0:0) | 1.958536 |
| Heme | 1.951376 |
| MG(20:4(5Z,8Z,11Z,14Z)/0:0/0:0) | 1.948045 |
| Pantetheine | 1.94656 |
| PC(15:0/18:2(9Z,12Z)) | 1.933874 |
| LysoPC(20:3(8Z,11Z,14Z)) | 1.93019 |
| LysoPS(16:0/0:0) | 1.92639 |
| 2-Arachidonylglycerol | 1.916232 |
| PI(16:0/18:1(9Z)) | 1.913343 |
| 1-Oleoylglycerophosphoinositol | 1.909143 |
| Stearic acid | 1.904901 |
| PC(18:2(9Z,12Z)/18:2(9Z,12Z)) | 1.903022 |
| MG(0:0/22:6(4Z,7Z,10Z,13Z,16Z,19Z)/0:0) | 1.882437 |
| LysoPE(20:4(5Z,8Z,11Z,14Z)/0:0) | 1.874989 |
| Bilirubin | 1.851473 |
| 2,4-Dihydroxyacetophenone 5-sulfate | 1.847354 |
| Propionylcarnitine | 1.843235 |
| Palmitic acid | 1.842019 |
| N-Acetyl-L-aspartic acid | 1.827562 |
| Alpha-Tocopherol | 1.81438 |
| TMAP | 1.805684 |
| SM(d18:2(4E,14Z)/23:0) | 1.785464 |
| Indoxyl sulfate | 1.785303 |
| Phenylacetylglutamine | 1.784154 |
| PC(18:0/22:4(7Z,10Z,13Z,16Z)) | 1.783442 |
| LysoPG(16:0/0:0) | 1.781061 |
| 2-Aminophenyl sulfate | 1.771089 |
| Inosinic acid | 1.762243 |
| LysoPE(0:0/20:5(5Z,8Z,11Z,14Z,17Z)) | 1.759282 |
| Hydroxyphenyllactic acid | 1.757934 |
| PG(18:0/18:1(9Z)) | 1.744254 |
| LysoPG(18:2(9Z,12Z)/0:0) | 1.71399 |
| LysoPC(15:0) | 1.704436 |
| PC(15:0/22:6(4Z,7Z,10Z,13Z,16Z,19Z)) | 1.687553 |
| PC(16:0/15:0) | 1.684349 |
| 5-KETE | 1.682082 |
| Hexadecanedioic acid | 1.67986 |
| L-Cysteine | 1.673012 |
| Metyrosine | 1.658246 |
| Mannose 6-phosphate | 1.657477 |
| MG(18:1(9Z)/0:0/0:0) | 1.655709 |
| PE(16:0/20:5(5Z,8Z,11Z,14Z,17Z)) | 1.644907 |
| Glycerylphosphorylethanolamine | 1.64297 |
| o-Tyrosine | 1.622398 |
| Cysteinylglycine | 1.622106 |
| Uridine diphosphate glucose | 1.620753 |
| PC(18:1(9Z)/22:6(4Z,7Z,10Z,13Z,16Z,19Z)) | 1.620355 |
| Propionyl-CoA | 1.619172 |
| Pentadecanoylcarnitine | 1.618872 |
| Arachidonic acid | 1.607151 |
| SM(d18:2(4E,14Z)/23:1(9Z)) | 1.606468 |
| SM(d16:1/23:0) | 1.605401 |
| N-Acetylmethionine | 1.589174 |
| 4-ethylphenylsulfate | 1.587381 |
| PC(18:0/22:6(4Z,7Z,10Z,13Z,16Z,19Z)) | 1.58675 |
| Docosanoylcarnitine | 1.577663 |
| Urea | 1.576979 |
| Betaine | 1.574468 |
| 1,2,3-Propanetricarboxylic acid | 1.570973 |
| PE(16:0/18:1(9Z)) | 1.568283 |
| Anserine | 1.567888 |
| O-methoxycatechol-O-sulphate | 1.566279 |
| D-Sedoheptulose 7-phosphate | 1.560279 |
| 2-Hydroxydecanoate | 1.558886 |
| Glycerophosphocholine | 1.555515 |
| N2,N2-Dimethylguanosine | 1.549701 |
| Deoxyinosine | 1.54114 |
| Glycerol | 1.538984 |
| 4-Pyridoxic acid | 1.535459 |
| Homocitrulline | 1.535077 |
| Creatinine | 1.532744 |
| PC(16:0/P-18:1(11Z)) | 1.53243 |
| Cinnamoylglycine | 1.530387 |
| S-Methylmethionine | 1.529329 |
| Phosphoenolpyruvic acid | 1.528237 |
| PE(P-16:0/20:4(5Z,8Z,11Z,14Z)) | 1.523778 |
| Methyl hexadecanoic acid | 1.523704 |
| PC(16:0/18:1(9Z)) | 1.523453 |
| PC(18:0/18:2(9Z,12Z)) | 1.521129 |
| MG(0:0/18:2(9Z,12Z)/0:0) | 1.510504 |
| N-Acetyltaurine | 1.50761 |
| PC(18:1(9Z)/18:2(9Z,12Z)) | 1.497065 |
| Adipic acid | 1.496009 |
| PE(18:0/22:6(4Z,7Z,10Z,13Z,16Z,19Z)) | 1.494923 |
| Vanilloylglycine | 1.494648 |
| Succinylcarnitine | 1.485676 |
| N6,N6,N6-Trimethyl-L-lysine | 1.484517 |
| Myristic acid | 1.483113 |
| 4-Vinylphenol sulfate | 1.482215 |
| N-Acetyl-D-glucosamine | 1.474287 |
| Octanoyl-CoA | 1.472023 |
| Prolyl-Alanine | 1.464545 |
| 3-[3-(Sulfooxy)phenyl]propanoic acid | 1.456643 |
| MG(0:0/14:0/0:0) | 1.450032 |
| L-Homoserine | 1.447346 |
| Thiamine pyrophosphate | 1.445759 |
| MG(18:2(9Z,12Z)/0:0/0:0) | 1.44476 |
| Pyrocatechol sulfate | 1.444203 |
| Ascorbic acid 3-sulfate | 1.441812 |
| Formylanthranilic acid | 1.439654 |
| 2-O-Methylascorbic acid | 1.432144 |
| Choline | 1.425467 |
| PC(P-16:0/22:6(4Z,7Z,10Z,13Z,16Z,19Z)) | 1.421116 |
| SM(d18:1/16:0) | 1.410082 |
| (R)-2-Hydroxycaprylic acid | 1.409539 |
| Alanyl-Leucine | 1.409301 |
| 2-Furoylglycine | 1.407416 |
| SM(d17:1/18:0) | 1.403628 |
| SM(d16:2(4E,8Z)/23:0) | 1.403035 |
| PC(18:0/18:1(9Z)) | 1.396164 |
| Docosatrienoic acid | 1.393515 |
| Palmitoleic acid | 1.392378 |
| Arachidonoylcholine | 1.390076 |
| DG(18:0/20:4(5Z,8Z,11Z,14Z)/0:0) | 1.383981 |
| Tyrosyl-Glycine | 1.382611 |
| Imidazolepropionic acid | 1.379174 |
| Suberic acid | 1.376929 |
| gamma-Carboxyglutamic acid | 1.373842 |
| SM(d18:1/23:0) | 1.373386 |
| (R)-3-Hydroxy-hexadecanoic acid | 1.373285 |
| Uridine diphosphate-N-acetylgalactosamine | 1.370776 |
| L-Octanoylcarnitine | 1.367916 |
| Valyl-Glutamine | 1.35668 |
| O-Phosphoethanolamine | 1.348961 |
| Lysyl-Alanine | 1.347353 |
| L-Cysteinylglycine disulfide | 1.343082 |
| Methyl beta-D-glucopyranoside | 1.338126 |
| 2-hydroxyglutaric acid | 1.33807 |
| 2,6-Dihydroxybenzoic acid | 1.337686 |
| Biliverdin | 1.335617 |
| Cyclic AMP | 1.329207 |
| Dihydroferulic acid 4-sulfate | 1.328412 |
| Leucyl-Alanine | 1.32688 |
| SAICAR | 1.326155 |
| LysoPC(16:1(9Z)) | 1.324937 |
| Azelaic acid | 1.32422 |
| Arachidyl carnitine | 1.322271 |
| LysoPE(20:3(11Z,14Z,17Z)/0:0) | 1.32002 |
| Oxalic acid | 1.31796 |
| PC(15:0/20:4(5Z,8Z,11Z,14Z)) | 1.316692 |
| 7-Methylguanine | 1.315126 |
| (R)-2-Hydroxyhexadecanoic acid | 1.31509 |
| PC(16:0/18:2(9Z,12Z)) | 1.306277 |
| Phenylalanyl-Glycine | 1.299556 |
| MG(0:0/16:0/0:0) | 1.297677 |
| 1-Methylhistamine | 1.297545 |
| Biopterin | 1.295791 |
| N8-Acetylspermidine | 1.294302 |
| Stearidonic acid | 1.288803 |
| Xanthosine | 1.279133 |
| N-alpha-Acetyl-L-citrulline | 1.278307 |
| (R)-3-hydroxybutyrylcarnitine | 1.276079 |
| 1-Methylnicotinamide | 1.273243 |
| N-Acetylhistidine | 1.27253 |
| MG(20:3(8Z,11Z,14Z)/0:0/0:0) | 1.272402 |
| Valyl-Leucine | 1.271641 |
| PC(16:0/18:3(9Z,12Z,15Z)) | 1.264887 |
| PC(20:0/20:4(5Z,8Z,11Z,14Z)) | 1.260431 |
| PC(16:0/18:0) | 1.259089 |
| N6-Carboxymethyllysine | 1.258565 |
| Palmitoylglycine | 1.257906 |
| Methionine sulfoxide | 1.257398 |
| SM(d18:1/14:0) | 1.254082 |
| MG(18:3(6Z,9Z,12Z)/0:0/0:0) | 1.250689 |
| 6-Oxopiperidine-2-carboxylic acid | 1.24475 |
| Methylimidazoleacetic acid | 1.240676 |
| PE(18:2(9Z,12Z)/18:2(9Z,12Z)) | 1.238719 |
| MG(0:0/18:1(9Z)/0:0) | 1.237025 |
| Hydroxybutyrylcarnitine | 1.232611 |
| PI(16:0/20:4(5Z,8Z,11Z,14Z)) | 1.223546 |
| PE(18:0/18:1(9Z)) | 1.219555 |
| Gly-Ile | 1.219444 |
| Cysteineglutathione disulfide | 1.216513 |
| Quinic acid | 1.214419 |
| N-Acetylneuraminic acid | 1.214276 |
| PE(P-18:0/22:6(4Z,7Z,10Z,13Z,16Z,19Z)) | 1.207619 |
| Undecanedioic acid | 1.206786 |
| 5-Dodecenoic acid | 1.202812 |
| LysoPI(18:2(9Z,12Z)/0:0) | 1.202691 |
| 9,10-DHOME | 1.194674 |
| 4-Hydroxynonenal | 1.183789 |
| PC(P-16:0/20:4(5Z,8Z,11Z,14Z)) | 1.183531 |
| Stearoylcarnitine | 1.177779 |
| LysoPC(22:4(7Z,10Z,13Z,16Z)) | 1.177036 |
| Leu-Gln | 1.175226 |
| Leu-Gly | 1.172532 |
| Vaccenic acid | 1.165935 |
| 3-hydroxyicosanoic Acid | 1.158985 |
| D-Ribose | 1.152659 |
| DG(18:2(9Z,12Z)/18:3(9Z,12Z,15Z)/0:0) | 1.149647 |
| Histamine | 1.144568 |
| Palmitoylcholine | 1.143605 |
| 4-Guanidinobutanoic acid | 1.140268 |
| 2-Methylbutyroylcarnitine | 1.132378 |
| Adenosine monophosphate | 1.129251 |
| Pi-Methylimidazoleacetic acid | 1.12589 |
| Hypoxanthine | 1.123047 |
| LysoPC(20:4(5Z,8Z,11Z,14Z)) | 1.119955 |
| Pantetheine 4'-phosphate | 1.110263 |
| L-Lyxonate | 1.108385 |
| Itaconic acid | 1.107701 |
| Imidazoleacetic acid riboside | 1.093246 |
| Thiamine monophosphate | 1.09171 |
| Glycylleucine | 1.09167 |
| Pentadecanoic acid | 1.09145 |
| DG(18:1(9Z)/18:2(9Z,12Z)/0:0) | 1.090562 |
| 2-Hydroxystearic acid | 1.088659 |
| PE(16:0/22:6(4Z,7Z,10Z,13Z,16Z,19Z)) | 1.087157 |
| Uridine 5'-monophosphate | 1.082173 |
| Hydroxypropanedioic acid | 1.078727 |
| N-stearoyl-sphingadienine | 1.07571 |
| Docosadienoate (22:2n6) | 1.072165 |
| Heptadecanoyl carnitine | 1.070804 |
| 3-Phosphoglyceric acid | 1.070627 |
| SM(d17:2(4E,8Z)/16:0) | 1.064996 |
| SM(d18:1/20:0) | 1.058347 |
| Spermine | 1.052334 |
| Palmityl-CoA | 1.048328 |
| PE(P-16:0/18:2(9Z,12Z)) | 1.047024 |
| Ureidosuccinic acid | 1.036781 |
| Valyl-Glycine | 1.032884 |
| Phenylalanyl-Alanine | 1.027468 |
| PE(18:1(9Z)/20:4(5Z,8Z,11Z,14Z)) | 1.02674 |
| 3-Aminopiperidine-2,6-dione | 1.017341 |
| Heptadecanoic acid | 1.0153 |
| Thr-Phe | 1.013361 |
| 1-Stearoylglycerophosphoserine | 1.010864 |
| Arachidic acid | 1.008623 |
| Niacinamide | 1.001117 |

**Table S7** Pathway Analysis results based on VIP score from OPLS-DA analysis using *Mus. musculus* database from MetaboAnalyst 5.0 (hypoxic vs normoxic group (N=10 for hypoxic and N=8 for normoxic group)

| Pathway Name | Match Status | p | -log(p) | Holm p | FDR | Impact |
| --- | --- | --- | --- | --- | --- | --- |
| Biosynthesis of unsaturated fatty acids | 8/36 | 8.057E-5 | 4.0938 | 0.0067678 | 0.0067678 | 0.13636 |
| Glycerophospholipid metabolism | 7/36 | 5.6537E-4 | 3.2477 | 0.046926 | 0.023746 | 0.35739 |
| Thiamine metabolism | 3/7 | 0.0022688 | 2.6442 | 0.18604 | 0.063527 | 0.66667 |
| Histidine metabolism | 4/16 | 0.0036754 | 2.4347 | 0.2977 | 0.077183 | 0.32786 |
| Purine metabolism | 8/66 | 0.0055263 | 2.2576 | 0.4421 | 0.092842 | 0.27239 |
| Pantothenate and CoA biosynthesis | 3/19 | 0.043605 | 1.3605 | 1.0 | 0.59181 | 0.27857 |
| Sphingolipid metabolism | 3/21 | 0.056363 | 1.249 | 1.0 | 0.59181 | 0.0142 |
| beta-Alanine metabolism | 3/21 | 0.056363 | 1.249 | 1.0 | 0.59181 | 0.05597 |
| Ascorbate and aldarate metabolism | 2/10 | 0.064212 | 1.1924 | 1.0 | 0.59931 | 0.0 |
| alpha-Linolenic acid metabolism | 2/13 | 0.10262 | 0.98877 | 1.0 | 0.7376 | 0.0 |
| Galactose metabolism | 3/27 | 0.10367 | 0.98433 | 1.0 | 0.7376 | 0.00228 |
| Glutathione metabolism | 3/28 | 0.11273 | 0.94795 | 1.0 | 0.7376 | 0.06023 |
| Glycosylphosphatidylinositol (GPI)-anchor biosynthesis | 2/14 | 0.11655 | 0.93349 | 1.0 | 0.7376 | 0.00399 |
| Nicotinate and nicotinamide metabolism | 2/15 | 0.13092 | 0.88298 | 1.0 | 0.7376 | 0.33246 |
| Porphyrin and chlorophyll metabolism | 3/30 | 0.13171 | 0.88036 | 1.0 | 0.7376 | 0.28772 |
| Glycine, serine and threonine metabolism | 3/34 | 0.17269 | 0.76274 | 1.0 | 0.80448 | 0.04655 |
| Linoleic acid metabolism | 1/5 | 0.19541 | 0.70905 | 1.0 | 0.80448 | 0.0 |
| Amino sugar and nucleotide sugar metabolism | 3/37 | 0.20553 | 0.68713 | 1.0 | 0.80448 | 0.21667 |
| Ether lipid metabolism | 2/20 | 0.20751 | 0.68296 | 1.0 | 0.80448 | 0.0 |
| Citrate cycle (TCA cycle) | 2/20 | 0.20751 | 0.68296 | 1.0 | 0.80448 | 0.05705 |
| Fatty acid elongation | 3/39 | 0.22818 | 0.64172 | 1.0 | 0.80448 | 0.03319 |
| Fatty acid degradation | 3/39 | 0.22818 | 0.64172 | 1.0 | 0.80448 | 0.06681 |
| Pyrimidine metabolism | 3/39 | 0.22818 | 0.64172 | 1.0 | 0.80448 | 0.18448 |
| Pyruvate metabolism | 2/22 | 0.23943 | 0.62082 | 1.0 | 0.80448 | 0.0114 |
| Pentose phosphate pathway | 2/22 | 0.23943 | 0.62082 | 1.0 | 0.80448 | 0.02775 |
| Propanoate metabolism | 2/23 | 0.2555 | 0.59261 | 1.0 | 0.82546 | 0.27158 |
| Lysine degradation | 2/25 | 0.28769 | 0.54107 | 1.0 | 0.87855 | 0.00469 |
| Taurine and hypotaurine metabolism | 1/8 | 0.29407 | 0.53155 | 1.0 | 0.87855 | 0.0 |
| Glycolysis / Gluconeogenesis | 2/26 | 0.30376 | 0.51747 | 1.0 | 0.87855 | 0.11308 |
| Fatty acid biosynthesis | 3/47 | 0.32233 | 0.49171 | 1.0 | 0.87855 | 0.01473 |
| Vitamin B6 metabolism | 1/9 | 0.32423 | 0.48915 | 1.0 | 0.87855 | 0.0 |
| Alanine, aspartate and glutamate metabolism | 2/28 | 0.33569 | 0.47406 | 1.0 | 0.88118 | 0.08654 |
| Arginine biosynthesis | 1/14 | 0.45701 | 0.34007 | 1.0 | 1.0 | 0.0 |
| Arachidonic acid metabolism | 2/36 | 0.45793 | 0.3392 | 1.0 | 1.0 | 0.33292 |
| Starch and sucrose metabolism | 1/15 | 0.4803 | 0.31849 | 1.0 | 1.0 | 0.01441 |
| Arginine and proline metabolism | 2/38 | 0.48656 | 0.31287 | 1.0 | 1.0 | 0.0 |
| Glycerolipid metabolism | 1/16 | 0.50261 | 0.29877 | 1.0 | 1.0 | 0.23676 |
| Valine, leucine and isoleucine degradation | 2/40 | 0.51425 | 0.28883 | 1.0 | 1.0 | 0.0936 |
| Pentose and glucuronate interconversions | 1/18 | 0.54443 | 0.26405 | 1.0 | 1.0 | 0.07812 |
| Fructose and mannose metabolism | 1/18 | 0.54443 | 0.26405 | 1.0 | 1.0 | 0.15735 |
| Phosphatidylinositol signaling system | 1/28 | 0.70689 | 0.15065 | 1.0 | 1.0 | 0.03736 |
| Inositol phosphate metabolism | 1/30 | 0.73173 | 0.13565 | 1.0 | 1.0 | 0.12939 |
| Glyoxylate and dicarboxylate metabolism | 1/32 | 0.7545 | 0.12234 | 1.0 | 1.0 | 0.0 |
| Cysteine and methionine metabolism | 1/33 | 0.76516 | 0.11625 | 1.0 | 1.0 | 0.09592 |
| Tryptophan metabolism | 1/41 | 0.83554 | 0.078033 | 1.0 | 1.0 | 0.00493 |
| Tyrosine metabolism | 1/42 | 0.84272 | 0.074314 | 1.0 | 1.0 | 0.0 |
| Primary bile acid biosynthesis | 1/46 | 0.8685 | 0.06123 | 1.0 | 1.0 | 0.0 |
| Aminoacyl-tRNA biosynthesis | 1/48 | 0.87978 | 0.055626 | 1.0 | 1.0 | 0.0 |

**Table S8** Significantly up and down biochemicals from Volcano plot with 2-Fold Change and p-value<0.05 in male mice, direction of comparison is from hypoxic to normoxic (N=5 for each hypoxic and normoxic group. Red fonts represent the upregulated metabolites, and the blue fonts represent the downregulated metabolites.

| Biochemicals | log2(FC) | -Log10 (P) | Pathway/ Metabolite Category |
| --- | --- | --- | --- |
| Pantetheine | 13.497 | 2.629 | Pantothenate and CoA Biosynthesis |
| Sorbitol | 13.232 | 1.3491 | Fructose and Mannose Degradation |
| SAICAR | 13.051 | 1.9493 | Purine Metabolism |
| N,N,N-Trimethyl-L-alanyl-L-proline betaine | 12.664 | 1.3806 | Dipeptide |
| Galactonic acid | 12.16 | 1.6526 | Galactitol and Galactonate Degradation |
| Glycerophosphocholine | 11.628 | 1.6095 | Phospholipid |
| Succinylcarnitine | 11.588 | 1.8987 | Acyl Carnitine |
| L-Homoserine | 11.466 | 1.7508 | Methionine Metabolism |
| PC(P-16:0/18:2(9Z,12Z)) | 11.165 | 1.3777 | Phospholipid |
| Docosanoylcarnitine | 10.781 | 1.5169 | Acyl Carnitine |
| PC(15:0/18:2(9Z,12Z)) | 10.652 | 1.4598 | Phospholipid |
| myo-Inositol | 10.538 | 3.0587 | Inositol Metabolism |
| Betaine | 10.448 | 1.3958 | Betaine Metabolism |
| PC(P-18:0/22:6(4Z,7Z,10Z,13Z,16Z,19Z)) | 10.446 | 1.6959 | Phospholipid |
| PC(18:0/18:2(9Z,12Z)) | 10.138 | 1.8011 | Phospholipid |
| PC(16:0/P-18:1(11Z)) | 10.112 | 1.7253 | Phospholipid |
| Glutamic acid, gamma-methyl ester | 9.4995 | 1.4527 | Glutamic acid and derivatives |
| L-Arabitol | 9.2328 | 1.344 | Sugar Alcohol |
| Urea | 7.4236 | 1.7868 | Urea Cycle |
| Glycerylphosphorylethanolamine | 7.2833 | 1.3043 | Phospholipid |
| Stearoylcarnitine | 6.9328 | 1.6915 | Acyl Carnitine |
| L-Cysteine | 5.5896 | 2.2174 | Methionine Metabolism |
| PC(16:0/15:0) | 5.5221 | 1.3345 | Phospholipid |
| PC(P-16:0/16:0) | 5.3792 | 1.5477 | Phospholipid |
| PC(18:0/22:5(4Z,7Z,10Z,13Z,16Z)) | 4.9476 | 2.2963 | Phospholipid |
| Adenosine monophosphate | 4.944 | 2.1141 | Purine Metabolism |
| Succinyladenosine | 4.941 | 1.8023 | Purine Nucleoside |
| 4-Trimethylammoniobutanoic acid | 4.3663 | 1.7112 | Carnitine Synthesis |
| PC(18:0/18:1(9Z)) | 4.0658 | 1.3891 | Phospholipid |
| Acetyl-CoA | 4.0512 | 1.9797 | Fatty acid metabolism |
| Cysteinylglycine | 3.9677 | 2.274 | Dipeptide |
| Heptadecanoyl carnitine | 3.884 | 1.5906 | Acyl Carnitine |
| Inosinic acid | 3.8745 | 1.6973 | Purine Metabolism |
| PC(18:2(9Z,12Z)/18:3(9Z,12Z,15Z)) | 3.6995 | 1.8308 | Phospholipid |
| Thiamine pyrophosphate | 3.5825 | 2.1263 | Thiamine Metabolism |
| Orotidine | 3.3748 | 1.5296 | Pyrimidine Metabolism |
| Arachidyl carnitine | 3.282 | 1.3902 | Acyl Carnitine |
| SM(d17:2(4E,8Z)/16:0) | 3.2476 | 1.4492 | Sphingolipid |
| PC(P-16:0/20:4(5Z,8Z,11Z,14Z)) | 3.1086 | 1.4971 | Phospholipid |
| Uridine 5'-monophosphate | 2.8874 | 1.3929 | Pyrimidine Metabolism |
| Guanosine monophosphate | 2.8655 | 1.4323 | Purine Metabolism |
| PE(P-16:0/20:4(5Z,8Z,11Z,14Z)) | 2.7112 | 1.3195 | Phospholipid |
| Alpha-Tocopherol | 2.6389 | 1.6576 | Active form of Vitamin E |
| AICAR | 2.1741 | 1.4138 | Purine Metabolism |
| N-Methyl-proline | 1.7265 | 1.3718 | Proline and derivatives |
| L-Cysteinylglycine disulfide | 1.0897 | 1.5796 | Dipeptide |
| Pyroglutamic acid | -1.9716 | 1.3723 | Glutathione Metabolism |
| Xylitol | -2.4925 | 1.3737 | Sugar alcohol |
| MG(0:0/22:6(4Z,7Z,10Z,13Z,16Z,19Z)/0:0) | -2.5082 | 1.3874 | Acyl Glycerol |
| Eicosapentaenoic acid | -2.6327 | 1.4741 | Fatty acid |
| LysoPG(18:1(9Z)/0:0) | -3.0807 | 1.6024 | Lysophospholipid |
| PE(O-16:1(1Z)/22:6(4Z,7Z,10Z,13Z,16Z,19Z)) | -3.5056 | 1.5261 | Phospholipid |
| L-Glutamic acid | -3.641 | 1.7814 | Glutamate Metabolism |
| Docosapentaenoic acid | -3.6607 | 2.4856 | Fatty Acid |
| N-Acetylvaline | -3.8633 | 1.7769 | N-acyl-alpha amino acids |
| 3 beta-Hydroxy-5-cholestenoate | -4.3007 | 1.8348 | primary bile acid biosynthesis pathway |
| MG(22:6(4Z,7Z,10Z,13Z,16Z,19Z)/0:0/0:0) | -4.6207 | 1.8038 | Acyl Glycerol |
| Cysteine-S-sulfate | -4.7517 | 1.4311 | Cysteine Biosynthesis |
| Deoxyinosine | -9.085 | 1.7714 | Purine Metabolism |
| LysoPE(0:0/18:0) | -10.633 | 2.7722 | Lysophospholipid |
| Docosahexaenoic acid | -11.321 | 1.4319 | Fatty Acid |
| 1-Arachidonoylglycerophosphoinositol | -11.332 | 1.7577 | Phospholipid |
| Spermine | -11.513 | 2.989 | Arginine and proline metabolism |
| 6,9,12,15,18,21-Tetracosahexaenoic acid | -11.527 | 1.3525 | Fatty acid |
| 2-Stearoylglycerophosphoinositol | -11.874 | 2.9806 | Phospholipid |
| Phosphoenolpyruvic acid | -13.415 | 1.6534 | Glycolysis |

**Table S9.** VIP score <1 from OPLS-DA for male hypoxic vs. normoxic group (N=5 for each hypoxic and normoxic group.

| Significant Features | V1P score |
| --- | --- |
| Linoleoyl ethanolamide | 2.173031 |
| myo-Inositol | 2.163824 |
| Spermine | 2.153513 |
| Propionyl-CoA | 2.075656 |
| Pantetheine | 2.06556 |
| Alanyl-Leucine | 2.062368 |
| Valyl-Glutamine | 2.009732 |
| Cysteinylglycine | 1.977338 |
| Thr-Phe | 1.969907 |
| Leucyl-Alanine | 1.968739 |
| L-Cysteine | 1.958416 |
| Thiamine pyrophosphate | 1.933917 |
| Adenosine monophosphate | 1.930132 |
| Stachydrine | 1.918095 |
| Gly-Ile | 1.888823 |
| Acetyl-CoA | 1.886572 |
| SAICAR | 1.868353 |
| Leu-Gly | 1.864052 |
| Succinyladenosine | 1.81831 |
| Deoxyinosine | 1.812665 |
| Histidinyl-Alanine | 1.812148 |
| Leu-Gln | 1.803149 |
| L-Glutamic acid | 1.803089 |
| N-Acetylvaline | 1.801511 |
| Urea | 1.79609 |
| Phenylalanyl-Alanine | 1.787044 |
| L-Homoserine | 1.774302 |
| 4-Trimethylammoniobutanoic acid | 1.772423 |
| Inosinic acid | 1.769635 |
| Alpha-Tocopherol | 1.749919 |
| Phosphoenolpyruvic acid | 1.741284 |
| Galactonic acid | 1.7401 |
| 4-Hydroxy-L-glutamic acid | 1.724605 |
| Phenylalanyl-Glycine | 1.724487 |
| L-Cysteinylglycine disulfide | 1.717979 |
| Tyrosyl-Glycine | 1.70566 |
| S-Methylmethionine | 1.679091 |
| Orotidine | 1.678857 |
| Pyrraline | 1.666809 |
| Isoleucyl-Glycine | 1.655691 |
| Cysteine-S-sulfate | 1.638384 |
| Guanosine monophosphate | 1.632781 |
| Glutamic acid, gamma-methyl ester | 1.63149 |
| Xylitol | 1.614922 |
| Uridine 5'-monophosphate | 1.614213 |
| Valyl-Glycine | 1.611429 |
| AICAR | 1.610866 |
| Betaine | 1.606228 |
| L-Arabitol | 1.602868 |
| Pyroglutamic acid | 1.597418 |
| Sucrose | 1.595586 |
| TMAP | 1.592622 |
| N-Methyl-proline | 1.590783 |
| L-Glutamine | 1.582647 |
| Sorbitol | 1.56953 |
| Glycylleucine | 1.561733 |
| 3-Sulfinoalanine | 1.55078 |
| 3-Aminopiperidine-2,6-dione | 1.543574 |
| (S)-NADHX | 1.534745 |
| Octanoyl-CoA | 1.530574 |
| Cyclic AMP | 1.526571 |
| Oxoglutaric acid | 1.523406 |
| Cytidine monophosphate | 1.518909 |
| Uridine diphosphate-N-acetylgalactosamine | 1.50336 |
| 4-Pyridoxic acid | 1.502883 |
| cis-Aconitic acid | 1.50244 |
| 2,4-Dihydroxyacetophenone 5-sulfate | 1.498306 |
| Ascorbic acid | 1.496417 |
| Metyrosine | 1.483358 |
| Palmitoylglycine | 1.473274 |
| Coenzyme A | 1.461611 |
| N-Acetylputrescine | 1.460355 |
| Anandamide (20:4, n-6) | 1.44851 |
| Hydroxyphenyllactic acid | 1.445448 |
| Ascorbic acid 3-sulfate | 1.432094 |
| Oleoylethanolamide | 1.426971 |
| Docosahexaenoyl Ethanolamide | 1.422182 |
| L-Carnitine | 1.420396 |
| N-Acetylserine | 1.417224 |
| Heme | 1.413938 |
| Aminoadipic acid | 1.408189 |
| L-Lactic acid | 1.403515 |
| Butyryl-CoA | 1.389373 |
| D-Fructose | 1.381258 |
| Uridine diphosphate glucose | 1.378444 |
| Galactose 1-phosphate | 1.372679 |
| Valyl-Leucine | 1.370385 |
| o-Tyrosine | 1.361753 |
| 1-Methylnicotinamide | 1.359226 |
| Cysteineglutathione disulfide | 1.353528 |
| 1,2,3-Propanetricarboxylic acid | 1.334206 |
| 3-Phosphoglyceric acid | 1.323066 |
| Adenylsuccinic acid | 1.309247 |
| Indoxyl sulfate | 1.308594 |
| D-Mannose | 1.307731 |
| N2,N2-Dimethylguanosine | 1.306506 |
| 4-ethylphenylsulfate | 1.301307 |
| Spermidine | 1.299927 |
| 1-Methylhistamine | 1.296144 |
| Pantetheine 4'-phosphate | 1.29433 |
| Nicotinamide ribotide | 1.292977 |
| Vanilloylglycine | 1.290735 |
| Histamine | 1.280845 |
| Methionine sulfone | 1.276322 |
| (S)C(S)S-S-Methylcysteine sulfoxide | 1.271264 |
| 7-Methylguanine | 1.265141 |
| Hexanoyl-CoA | 1.264267 |
| Creatinine | 1.262849 |
| Methyl beta-D-glucopyranoside | 1.260387 |
| N-Carboxyethyl-g-aminobutyric acid | 1.259498 |
| FAD | 1.24982 |
| Methionine sulfoxide | 1.247448 |
| N-Acetyl-L-aspartic acid | 1.245476 |
| 3-Hydroxyisovaleric acid | 1.238614 |
| N-Acetyl-L-tyrosine | 1.238236 |
| O-methoxycatechol-O-sulphate | 1.233392 |
| N-Acetylasparagine | 1.232468 |
| N-Acetyl-glucosamine 1-phosphate | 1.220449 |
| D-Ribose | 1.219817 |
| Tryptophan 2-C-mannoside | 1.211664 |
| Alpha-ketoisovaleric acid | 1.201428 |
| Adenosine | 1.200074 |
| N-alpha-Acetyl-L-citrulline | 1.196245 |
| Homocitrulline | 1.190821 |
| Sedoheptulose | 1.189626 |
| Cytosine | 1.186357 |
| 1-Methylinosine | 1.184787 |
| Guanosine | 1.179158 |
| Hypoxanthine | 1.178017 |
| Ribothymidine | 1.173761 |
| N-Acetylneuraminic acid | 1.171668 |
| Biopterin | 1.169594 |
| 3-Methoxytyrosine | 1.169023 |
| Argininosuccinic acid | 1.164034 |
| Palmityl-CoA | 1.161135 |
| Oxalic acid | 1.147471 |
| N-Acetyl-D-glucosamine | 1.14453 |
| Pi-Methylimidazoleacetic acid | 1.143896 |
| Bilirubin | 1.140391 |
| 5'-Methylthioadenosine | 1.13761 |
| Hypotaurine | 1.126489 |
| Prolyl-Alanine | 1.124845 |
| Palmitoylethanolamide | 1.118959 |
| Methylimidazoleacetic acid | 1.117732 |
| 6-Oxopiperidine-2-carboxylic acid | 1.117455 |
| Formylanthranilic acid | 1.10609 |
| N-Acetyl-1-methylhistidine | 1.102244 |
| L-gamma-glutamyl-L-isoleucine | 1.099681 |
| Urocanic acid | 1.091136 |
| L-Lyxonate | 1.087057 |
| L-Histidine | 1.08656 |
| Lysyl-Alanine | 1.082877 |
| Citicoline | 1.080166 |
| Thyroxine | 1.07913 |
| 5,6-Dihydrouridine | 1.077459 |
| Allantoin | 1.077175 |
| S-Adenosylhomocysteine | 1.073738 |
| Glycylproline | 1.063787 |
| Indolelactic acid | 1.06349 |
| Sphinganine | 1.059428 |
| N6-Carbamoyl-L-threonyladenosine | 1.0567 |
| O-Sulfotyrosine | 1.055054 |
| N-Acetylglutamine | 1.049746 |
| gamma-Glutamylglutamic acid | 1.047941 |
| Quinol sulfate | 1.045591 |
| Dihydroferulic acid 4-sulfate | 1.042692 |
| Adenosine 3',5'-diphosphate | 1.030587 |
| Adenosine 3',5'-bisphosphate | 1.030587 |
| L-Aspartic acid | 1.025926 |
| Pyridoxamine 5'-phosphate | 1.025344 |
| Nicotinamide N-oxide | 1.024507 |
| Arabinonic acid | 1.023123 |
| Fructose 1,6-bisphosphate | 1.019519 |
| Hydroxypropanedioic acid | 1.017236 |
| ADP-ribose | 1.007294 |

**Table S10**. Pathway Analysis results for male group based on VIP score from OPLS-DA analysis using *Mus. musculus* database from MetaboAnalyst 5.0 (hypoxic vs normoxic male group (N=5 for Hypoxic and N=5 for normoxic group)

| Pathway Name | Match Status | p | -log(p) | Holm p | FDR | Impact |
| --- | --- | --- | --- | --- | --- | --- |
| Purine metabolism | 14/66 | 2.3555E-6 | 5.6279 | 1.9787E-4 | 1.9787E-4 | 0.40435 |
| Histidine metabolism | 7/16 | 5.4983E-6 | 5.2598 | 4.5636E-4 | 2.3093E-4 | 0.62294 |
| Pantothenate and CoA biosynthesis | 7/19 | 2.1372E-5 | 4.6702 | 0.0017525 | 5.9841E-4 | 0.48571 |
| Arginine biosynthesis | 6/14 | 3.1875E-5 | 4.4965 | 0.0025819 | 6.6938E-4 | 0.2335 |
| Glutathione metabolism | 7/28 | 3.4529E-4 | 3.4618 | 0.027623 | 0.0048341 | 0.09417 |
| Alanine, aspartate and glutamate metabolism | 7/28 | 3.4529E-4 | 3.4618 | 0.027623 | 0.0048341 | 0.71234 |
| beta-Alanine metabolism | 6/21 | 4.3151E-4 | 3.365 | 0.033658 | 0.0051782 | 0.11194 |
| D-Glutamine and D-glutamate metabolism | 3/6 | 0.0023007 | 2.6381 | 0.17716 | 0.024158 | 0.5 |
| Citrate cycle (TCA cycle) | 5/20 | 0.0025982 | 2.5853 | 0.19746 | 0.02425 | 0.20232 |
| Butanoate metabolism | 4/15 | 0.0056087 | 2.2511 | 0.42065 | 0.045653 | 0.11111 |
| Taurine and hypotaurine metabolism | 3/8 | 0.0059784 | 2.2234 | 0.4424 | 0.045653 | 0.57142 |
| Glycolysis / Gluconeogenesis | 5/26 | 0.0086579 | 2.0626 | 0.63203 | 0.060605 | 0.22679 |
| Galactose metabolism | 5/27 | 0.010208 | 1.9911 | 0.73495 | 0.065957 | 0.07496 |
| Fatty acid degradation | 6/39 | 0.012403 | 1.9065 | 0.88059 | 0.074416 | 0.43629 |
| Glyoxylate and dicarboxylate metabolism | 5/32 | 0.020845 | 1.681 | 1.0 | 0.11673 | 0.03175 |
| Pyruvate metabolism | 4/22 | 0.022885 | 1.6404 | 1.0 | 0.12015 | 0.16537 |
| Nitrogen metabolism | 2/6 | 0.033857 | 1.4704 | 1.0 | 0.16729 | 0.0 |
| Nicotinate and nicotinamide metabolism | 3/15 | 0.037499 | 1.426 | 1.0 | 0.17499 | 0.16974 |
| Fatty acid elongation | 5/39 | 0.045119 | 1.3456 | 1.0 | 0.19256 | 0.30178 |
| Thiamine metabolism | 2/7 | 0.045848 | 1.3387 | 1.0 | 0.19256 | 0.66667 |
| Pentose and glucuronate interconversions | 3/18 | 0.06027 | 1.2199 | 1.0 | 0.24108 | 0.25 |
| Vitamin B6 metabolism | 2/9 | 0.073566 | 1.1333 | 1.0 | 0.28089 | 0.04902 |
| Cysteine and methionine metabolism | 4/33 | 0.083918 | 1.0761 | 1.0 | 0.30648 | 0.16524 |
| Ascorbate and aldarate metabolism | 2/10 | 0.088984 | 1.0507 | 1.0 | 0.31144 | 0.0 |
| Aminoacyl-tRNA biosynthesis | 5/48 | 0.094135 | 1.0263 | 1.0 | 0.31629 | 0.0 |
| Propanoate metabolism | 3/23 | 0.10917 | 0.96188 | 1.0 | 0.35271 | 0.28427 |
| Amino sugar and nucleotide sugar metabolism | 4/37 | 0.11639 | 0.93407 | 1.0 | 0.36212 | 0.15197 |
| Arginine and proline metabolism | 4/38 | 0.12527 | 0.90214 | 1.0 | 0.37582 | 0.14347 |
| Lysine degradation | 3/25 | 0.13198 | 0.87948 | 1.0 | 0.37641 | 0.14554 |
| Pyrimidine metabolism | 4/39 | 0.13443 | 0.87149 | 1.0 | 0.37641 | 0.16327 |
| Valine, leucine and isoleucine degradation | 4/40 | 0.14386 | 0.84205 | 1.0 | 0.38982 | 0.1328 |
| Starch and sucrose metabolism | 2/15 | 0.17654 | 0.75316 | 1.0 | 0.46341 | 0.06846 |
| Riboflavin metabolism | 1/4 | 0.18953 | 0.72232 | 1.0 | 0.48208 | 0.0 |
| Porphyrin and chlorophyll metabolism | 3/30 | 0.19513 | 0.70968 | 1.0 | 0.48208 | 0.21307 |
| Synthesis and degradation of ketone bodies | 1/5 | 0.23108 | 0.63624 | 1.0 | 0.54551 | 0.0 |
| Fructose and mannose metabolism | 2/18 | 0.23379 | 0.63117 | 1.0 | 0.54551 | 0.08454 |
| Sphingolipid metabolism | 2/21 | 0.29203 | 0.53458 | 1.0 | 0.66298 | 0.15416 |
| Pentose phosphate pathway | 2/22 | 0.31139 | 0.5067 | 1.0 | 0.68833 | 0.0 |
| Valine, leucine and isoleucine biosynthesis | 1/8 | 0.34352 | 0.46405 | 1.0 | 0.73989 | 0.0 |
| Inositol phosphate metabolism | 2/30 | 0.45973 | 0.3375 | 1.0 | 0.96543 | 0.12939 |
| Glycosylphosphatidylinositol (GPI)-anchor biosynthesis | 1/14 | 0.52194 | 0.28238 | 1.0 | 1.0 | 0.0 |
| Glycine, serine and threonine metabolism | 2/34 | 0.52697 | 0.27821 | 1.0 | 1.0 | 0.04655 |
| Terpenoid backbone biosynthesis | 1/18 | 0.61334 | 0.2123 | 1.0 | 1.0 | 0.0 |
| Tryptophan metabolism | 2/41 | 0.63065 | 0.20021 | 1.0 | 1.0 | 0.00493 |
| Fatty acid biosynthesis | 2/47 | 0.70495 | 0.15184 | 1.0 | 1.0 | 0.00213 |
| Phosphatidylinositol signaling system | 1/28 | 0.77309 | 0.11177 | 1.0 | 1.0 | 0.03736 |
| Glycerophospholipid metabolism | 1/36 | 0.85226 | 0.06943 | 1.0 | 1.0 | 0.01925 |
| Biosynthesis of unsaturated fatty acids | 1/36 | 0.85226 | 0.06943 | 1.0 | 1.0 | 0.13636 |
| Tyrosine metabolism | 1/42 | 0.89308 | 0.049111 | 1.0 | 1.0 | 0.0 |

**Table S11** Significantly up and down biochemicals from Volcano plot with 2-Fold Change and p-value<0.05 in female mice, direction of comparison is from hypoxic to normoxic (N=5 for hypoxic group and N=3 for normoxic group). Red fonts represent the upregulated metabolites and the blue fonts represent the downregulated metabolites.

| Biochemicals | log2(FC) | -Log10 (P) | Pathway/ Metabolite Category |
| --- | --- | --- | --- |
| Sphingosine | 11.901 | 2.2074 | Sphingosine |
| Anserine | 11.883 | 1.9932 | Histidine Metabolism |
| D-Mannose | 11.706 | 1.5803 | Fructose and Mannose Degradation |
| PC(18:2(9Z,12Z)/18:3(9Z,12Z,15Z)) | 11.619 | 1.5488 | Phospholipid |
| Sedoheptulose | 11.449 | 2.0035 | Pentose Phosphate Pathway |
| Xanthurenic acid | 11.373 | 2.1632 | Tryptophan Metabolism |
| Cysteine-S-sulfate | 10.972 | 1.5902 | Cysteine Biosynthesis |
| Gulonic acid | 10.593 | 2.6853 | Medium-chain hydroxy acids and derivatives |
| PG(18:0/18:1(9Z)) | 8.2636 | 1.3784 | Phospholipid |
| PC(P-18:0/22:6(4Z,7Z,10Z,13Z,16Z,19Z)) | 2.8141 | 1.3569 | Phospholipid |
| Thyroxine | -1.8518 | 1.5304 | Thyroxine Synthesis |
| Urea | -2.6185 | 1.5779 | Urea Cycle |
| LysoPC(20:4(5Z,8Z,11Z,14Z)) | -2.9906 | 1.6002 | Lysohospholipid |
| LysoPE(20:4(5Z,8Z,11Z,14Z)/0:0) | -3.0178 | 1.7957 | Lysohospholipid |
| LysoPG(16:0/0:0) | -3.0675 | 1.7063 | Lysohospholipid |
| Sorbitol | -3.3404 | 2.1817 | Fructose and Mannose Degradation |
| LysoPE(0:0/18:3(9Z,12Z,15Z)) | -3.3476 | 1.5456 | Lysohospholipid |
| 2-Palmitoylglycerophosphocholine | -3.5822 | 2.2991 | Phospholipid |
| Imidazolepropionic acid | -4.1971 | 2.7825 | Histidine Metabolism |
| D-Fructose | -4.8324 | 1.5258 | Fructose and Mannose Degradation |
| Uric acid | -6.2754 | 1.3179 | Purine Metabolism |
| Cysteinylglycine | -8.0666 | 1.4832 | Dipeptide |
| Phenylacetylglycine | -9.0756 | 1.9003 | Dipeptide |
| PC(P-16:0/16:0) | -12.14 | 3.0595 | Phospholipid |
| ADP | -12.698 | 2.2603 | Purine Metabolism |

**Table S12.** VIP score <1 from OPLS-DA for female Hypoxic vs Normoxic group (N=5 for hypoxic group and N=3 for normoxic group).

| Significant Features | VIP score |
| --- | --- |
| Gulonic acid | 2.518866 |
| Imidazolepropionic acid | 2.49154 |
| 2-Furoylglycine | 2.480398 |
| ADP | 2.402647 |
| Sorbitol | 2.352655 |
| Xanthurenic acid | 2.351992 |
| Sedoheptulose | 2.335529 |
| Anserine | 2.282943 |
| Phenylacetylglycine | 2.236115 |
| D-Mannose | 2.161513 |
| Cysteine-S-sulfate | 2.09161 |
| 2,6-Dihydroxybenzoic acid | 2.086647 |
| D-Fructose | 2.082485 |
| Cysteinylglycine | 2.057449 |
| 2-Aminophenyl sulfate | 2.035585 |
| Thyroxine | 2.033281 |
| Urea | 2.028527 |
| Docosahexaenoyl Ethanolamide | 2.021169 |
| Cinnamoylglycine | 1.995791 |
| Betonicine | 1.961969 |
| Uric acid | 1.951775 |
| Symmetric dimethylarginine | 1.939027 |
| Phenylalanyl-Alanine | 1.936151 |
| Quinol sulfate | 1.920663 |
| Histidinyl-Alanine | 1.885135 |
| L-Carnitine | 1.849682 |
| L-Targinine | 1.838413 |
| Pyrocatechol sulfate | 1.767922 |
| Xylitol | 1.753102 |
| Putrescine | 1.743472 |
| Oleoylethanolamide | 1.733974 |
| N-Acetylmethionine | 1.727073 |
| D-Lysine | 1.722775 |
| myo-Inositol | 1.71233 |
| Phenylacetylglutamine | 1.694233 |
| L-Arginine | 1.686809 |
| N-Alpha-acetyllysine | 1.679419 |
| N-Acetylleucine | 1.671967 |
| Uridine | 1.666364 |
| L-Glutamine | 1.656955 |
| Succinyladenosine | 1.62805 |
| Thiamine | 1.627584 |
| Fructosyllysine | 1.625707 |
| L-Phenylalanine | 1.624793 |
| 1-Methylinosine | 1.607037 |
| Isoleucyl-Glycine | 1.577069 |
| N-Acetylputrescine | 1.573165 |
| N-Carboxyethyl-g-aminobutyric acid | 1.567802 |
| L-Lactic acid | 1.563409 |
| Glutamic acid, gamma-methyl ester | 1.561504 |
| Saccharopine | 1.548004 |
| N-Acetylvaline | 1.541079 |
| 2-Keto-glutaramic acid | 1.537384 |
| Leu-Gly | 1.534312 |
| Gluconic acid | 1.527186 |
| Ureidopropionic acid | 1.518293 |
| L-Cystine | 1.514234 |
| Maltotetraose | 1.504647 |
| Citrulline | 1.502741 |
| Hydrocinnamic acid | 1.501027 |
| Mannose 6-phosphate | 1.492614 |
| L-Threonine | 1.490992 |
| Acetyl-CoA | 1.488881 |
| Inosine | 1.486632 |
| (S)-NADHX | 1.468215 |
| 4-Methylcatechol 1-sulfate | 1.467725 |
| Guanidoacetic acid | 1.462714 |
| Guanosine monophosphate | 1.459814 |
| L-Cysteinylglycine disulfide | 1.45596 |
| Imidazoleacetic acid riboside | 1.455667 |
| Sphinganine | 1.439877 |
| Galactonic acid | 1.439014 |
| Hexanoyl-CoA | 1.437433 |
| L-Leucine | 1.437114 |
| Adenylsuccinic acid | 1.429741 |
| (S)-3-Hydroxybutyric acid | 1.425961 |
| Adenosine monophosphate | 1.424854 |
| Prolylhydroxyproline | 1.422756 |
| 5-Aminopentanoic acid | 1.419116 |
| Pantetheine | 1.370612 |
| L-Serine | 1.36972 |
| Pyroglutamic acid | 1.362814 |
| gamma-Carboxyglutamic acid | 1.359197 |
| Maltopentaose | 1.356478 |
| Coenzyme A | 1.350232 |
| Indole-3-propionic acid | 1.348616 |
| Myo-inositol 1-phosphate | 1.343158 |
| Heme | 1.342563 |
| L-Histidine | 1.332702 |
| Orotic acid | 1.318658 |
| Leucyl-Alanine | 1.315882 |
| 2,3-Dihydroxy-5-methylthio-4-pentenoic acid | 1.31276 |
| Cytidine | 1.312042 |
| Phenylpropionylglycine | 1.306618 |
| Asp-Asp | 1.302598 |
| L-Glutamic acid | 1.298049 |
| Biliverdin | 1.295732 |
| Valyl-Glycine | 1.293511 |
| Ornithine | 1.292655 |
| Cytidine monophosphate | 1.292297 |
| Bilirubin | 1.290615 |
| Phenylalanyl-Glycine | 1.284876 |
| L-Isoleucine | 1.271268 |
| Propionyl-CoA | 1.267346 |
| 4-Vinylphenol sulfate | 1.263969 |
| Nicotinamide ribotide | 1.261329 |
| D-Sedoheptulose 7-phosphate | 1.257077 |
| Argininic acid | 1.238146 |
| Glycerol | 1.21923 |
| Glycylleucine | 1.219103 |
| N-Acetyl-L-alanine | 1.217087 |
| Niacinamide | 1.207589 |
| cis-Aconitic acid | 1.201529 |
| Sucrose | 1.196939 |
| L-Alanine | 1.190899 |
| Ophthalmic acid | 1.186192 |
| 3-[3-(Sulfooxy)phenyl]propanoic acid | 1.176068 |
| L-Asparagine | 1.174288 |
| N2-gamma-Glutamylglutamine | 1.170079 |
| Thr-Phe | 1.167896 |
| Succinic acid | 1.16548 |
| 3-Methylglutaconic acid | 1.164447 |
| Orotidine | 1.151687 |
| L-Arabitol | 1.120673 |
| 3-Sulfinoalanine | 1.12039 |
| Thymidine | 1.115638 |
| Capryloylglycine | 1.113697 |
| Pyridoxal 5'-phosphate | 1.109885 |
| Homocitrulline | 1.099951 |
| Linoleoyl ethanolamide | 1.094519 |
| 4-Trimethylammoniobutanoic acid | 1.094483 |
| Aminovaleric acid betaine | 1.092853 |
| N-Acetylserine | 1.088376 |
| N-Acetyl-L-aspartic acid | 1.08519 |
| N-Acetylglutamine | 1.080061 |
| D-Glucose | 1.077347 |
| Uridine 5'-diphosphate | 1.071937 |
| L-Valine | 1.069984 |
| Imidazolelactic acid | 1.069874 |
| 2-Methylbutyryl-CoA | 1.064422 |
| S-(methyl)glutathione | 1.058351 |
| Pyrraline | 1.056409 |
| Glyceric acid | 1.055366 |
| Deoxyuridine | 1.054777 |
| N-Acetyltaurine | 1.053159 |
| L-Proline | 1.051769 |
| (S)C(S)S-S-Methylcysteine sulfoxide | 1.051587 |
| L-Methionine | 1.050638 |
| 4-Acetamidobutanoic acid | 1.050038 |
| Citicoline | 1.037937 |
| S-Methyl-L-cysteine | 1.034008 |
| L-Tyrosine | 1.026857 |
| Thioproline | 1.026007 |
| Inosinic acid | 1.025364 |
| Ursodeoxycholic acid | 1.024776 |
| Acetylphosphate | 1.022518 |
| Indoxyl sulfate | 1.020713 |
| Deoxyadenosine | 1.006333 |
| Tyrosyl-Glycine | 1.005383 |
| Glycine | 1.004558 |

**Table S13** Pathway Analysis results for female group based on VIP score from OPLS-DA analysis using *mus. musculus* database from MetaboAnalyst 5.0 (Hypoxic vs Normoxic male group (N=5 for Hypoxic and N=3for Normoxic group)

| Pathway Name | Match Status | p | -log(p) | Holm p | FDR | Impact |
| --- | --- | --- | --- | --- | --- | --- |
| Aminoacyl-tRNA biosynthesis | 16/48 | 1.3653E-9 | 8.8648 | 1.1468E-7 | 1.1468E-7 | 0.16667 |
| Pyrimidine metabolism | 10/39 | 3.1931E-5 | 4.4958 | 0.0026502 | 0.0013411 | 0.3121 |
| Arginine biosynthesis | 6/14 | 5.6605E-5 | 4.2471 | 0.0046416 | 0.0015849 | 0.48223 |
| Alanine, aspartate and glutamate metabolism | 8/28 | 9.116E-5 | 4.0402 | 0.007384 | 0.0019144 | 0.41907 |
| Glyoxylate and dicarboxylate metabolism | 8/32 | 2.5593E-4 | 3.5919 | 0.020475 | 0.0042997 | 0.25927 |
| Valine, leucine and isoleucine biosynthesis | 4/8 | 5.5676E-4 | 3.2543 | 0.043984 | 0.0076841 | 0.0 |
| Glutathione metabolism | 7/28 | 6.4034E-4 | 3.1936 | 0.049947 | 0.0076841 | 0.17947 |
| Arginine and proline metabolism | 8/38 | 8.9936E-4 | 3.0461 | 0.069251 | 0.0094433 | 0.48789 |
| Purine metabolism | 10/66 | 0.0029864 | 2.5249 | 0.22697 | 0.027873 | 0.29317 |
| beta-Alanine metabolism | 5/21 | 0.005055 | 2.2963 | 0.37912 | 0.042462 | 0.16045 |
| Phenylalanine, tyrosine and tryptophan biosynthesis | 2/4 | 0.017539 | 1.756 | 1.0 | 0.13371 | 1.0 |
| Pantothenate and CoA biosynthesis | 4/19 | 0.019101 | 1.7189 | 1.0 | 0.13371 | 0.24643 |
| Valine, leucine and isoleucine degradation | 6/40 | 0.022067 | 1.6562 | 1.0 | 0.14221 | 0.12028 |
| Porphyrin and chlorophyll metabolism | 5/30 | 0.023702 | 1.6252 | 1.0 | 0.14221 | 0.28772 |
| Phenylalanine metabolism | 3/12 | 0.026357 | 1.5791 | 1.0 | 0.1476 | 0.35714 |
| Cysteine and methionine metabolism | 5/33 | 0.034567 | 1.4613 | 1.0 | 0.18 | 0.14814 |
| Glycine, serine and threonine metabolism | 5/34 | 0.038776 | 1.4114 | 1.0 | 0.18 | 0.54841 |
| Nitrogen metabolism | 2/6 | 0.040714 | 1.3903 | 1.0 | 0.18 | 0.0 |
| D-Glutamine and D-glutamate metabolism | 2/6 | 0.040714 | 1.3903 | 1.0 | 0.18 | 0.5 |
| Butanoate metabolism | 3/15 | 0.048241 | 1.3166 | 1.0 | 0.20261 | 0.0 |
| Histidine metabolism | 3/16 | 0.057017 | 1.244 | 1.0 | 0.22807 | 0.27049 |
| Galactose metabolism | 4/27 | 0.061403 | 1.2118 | 1.0 | 0.23445 | 0.03888 |
| Pentose and glucuronate interconversions | 3/18 | 0.076645 | 1.1155 | 1.0 | 0.27992 | 0.25 |
| Citrate cycle (TCA cycle) | 3/20 | 0.098829 | 1.0051 | 1.0 | 0.3459 | 0.11944 |
| Ascorbate and aldarate metabolism | 2/10 | 0.10552 | 0.97665 | 1.0 | 0.35456 | 0.0 |
| Pentose phosphate pathway | 3/22 | 0.12328 | 0.9091 | 1.0 | 0.38354 | 0.07487 |
| Pyruvate metabolism | 3/22 | 0.12328 | 0.9091 | 1.0 | 0.38354 | 0.15397 |
| Propanoate metabolism | 3/23 | 0.13626 | 0.86563 | 1.0 | 0.40878 | 0.27919 |
| Lysine degradation | 3/25 | 0.16353 | 0.7864 | 1.0 | 0.47368 | 0.05164 |
| Glycolysis / Gluconeogenesis | 3/26 | 0.17774 | 0.75021 | 1.0 | 0.49768 | 0.0419 |
| Nicotinate and nicotinamide metabolism | 2/15 | 0.20589 | 0.68635 | 1.0 | 0.55791 | 0.22588 |
| Glycerolipid metabolism | 2/16 | 0.22716 | 0.64366 | 1.0 | 0.5963 | 0.33022 |
| Inositol phosphate metabolism | 3/30 | 0.23752 | 0.6243 | 1.0 | 0.60459 | 0.15806 |
| Synthesis and degradation of ketone bodies | 1/5 | 0.25239 | 0.59792 | 1.0 | 0.62356 | 0.0 |
| Fructose and mannose metabolism | 2/18 | 0.27008 | 0.56851 | 1.0 | 0.64819 | 0.19048 |
| Sphingolipid metabolism | 2/21 | 0.33426 | 0.47591 | 1.0 | 0.75985 | 0.15416 |
| Thiamine metabolism | 1/7 | 0.3347 | 0.47535 | 1.0 | 0.75985 | 0.0 |
| Taurine and hypotaurine metabolism | 1/8 | 0.37242 | 0.42897 | 1.0 | 0.81769 | 0.28571 |
| Fatty acid degradation | 3/39 | 0.37964 | 0.42062 | 1.0 | 0.81769 | 0.328 |
| Ubiquinone and other terpenoid-quinone biosynthesis | 1/9 | 0.40803 | 0.38931 | 1.0 | 0.83597 | 0.0 |
| Vitamin B6 metabolism | 1/9 | 0.40803 | 0.38931 | 1.0 | 0.83597 | 0.2549 |
| Phosphatidylinositol signaling system | 2/28 | 0.47583 | 0.32255 | 1.0 | 0.95166 | 0.06789 |
| Starch and sucrose metabolism | 1/15 | 0.58341 | 0.23403 | 1.0 | 1.0 | 0.05405 |
| Amino sugar and nucleotide sugar metabolism | 2/37 | 0.62896 | 0.20138 | 1.0 | 1.0 | 0.08137 |
| Terpenoid backbone biosynthesis | 1/18 | 0.65071 | 0.18661 | 1.0 | 1.0 | 0.0 |
| Fatty acid elongation | 2/39 | 0.65796 | 0.1818 | 1.0 | 1.0 | 0.26859 |
| Selenocompound metabolism | 1/20 | 0.68949 | 0.16147 | 1.0 | 1.0 | 0.0 |
| Tyrosine metabolism | 2/42 | 0.69808 | 0.15609 | 1.0 | 1.0 | 0.13972 |
| Glycerophospholipid metabolism | 1/36 | 0.8796 | 0.055717 | 1.0 | 1.0 | 0.01925 |
| Tryptophan metabolism | 1/41 | 0.91064 | 0.040651 | 1.0 | 1.0 | 0.0 |
| Primary bile acid biosynthesis | 1/46 | 0.93376 | 0.029767 | 1.0 | 1.0 | 0.02239 |
| Fatty acid biosynthesis | 1/47 | 0.93761 | 0.027977 | 1.0 | 1.0 | 0.00213 |

**TableS14.** Covariate Analysis from Two-Way ANOVA with p-value <0.05

| Metabolites | Log_2_FC | AveExpr | t | p.value | adj.p.val. | B |
| --- | --- | --- | --- | --- | --- | --- |
| Galactonic acid | -0.7598 | -0.11475 | -3.3845 | 0.003419 | 0.96375 | -3.4429 |
| Pantetheine | -0.6497 | -0.05422 | -3.1655 | 0.005511 | 0.96375 | -3.5592 |
| Linoleoyl ethanolamide | 0.2782 | 0.081791 | 3.0188 | 0.007568 | 0.96375 | -3.6385 |
| 4-Trimethylammoniobutanoic acid | -0.28361 | -0.01143 | -2.7318 | 0.013961 | 0.96375 | -3.7954 |
| Heme | 0.6296 | -0.11996 | 2.6366 | 0.017051 | 0.96375 | -3.8476 |
| Oleoylethanolamide | 0.24435 | 0.028038 | 2.5936 | 0.018651 | 0.96375 | -3.8712 |
| Quinol sulfate | -0.9197 | -0.03651 | -2.5261 | 0.021457 | 0.96375 | -3.9083 |
| L-Cysteine | -0.48333 | 0.06662 | -2.4324 | 0.026016 | 0.96375 | -3.9595 |
| Thyroxine | 0.23524 | -0.14694 | 2.3557 | 0.030415 | 0.96375 | -4.0013 |
| Orotidine | -0.1905 | 0.016755 | -2.3538 | 0.03053 | 0.96375 | -4.0023 |
| Stachydrine | -0.29845 | -0.11751 | -2.3179 | 0.032826 | 0.96375 | -4.0218 |
| 2,4-Dihydroxyacetophenone 5-sulfate | -0.95821 | -0.32093 | -2.2496 | 0.037642 | 0.96375 | -4.0587 |
| Uridine diphosphate glucose | -0.65735 | 0.13215 | -2.1211 | 0.048516 | 0.96375 | -4.1274 |
| TMAP | -0.30745 | 0.10452 | -2.113 | 0.049283 | 0.96375 | -4.1316 |
| Indoxyl sulfate | -0.55711 | 0.015279 | -2.1119 | 0.049389 | 0.96375 | -4.1322 |
| Inosinic acid | -0.59151 | -0.13209 | -2.1105 | 0.049527 | 0.96375 | -4.133 |

**Table S15** Biomarkers identified using biomarker discovery feature from MetaboAnalyst 5.0

| Name | AUC | T-tests |
| --- | --- | --- |
| Linoleoyl ethanolamide | 0.9 | 0.0040637 |
| Glutamic acid gamma-methyl ester | 0.8875 | 0.0038434 |
| L-Carnitine | 0.875 | 0.014076 |
| Galactonic acid | 0.875 | 0.0037443 |
| 4-Trimethylammoniobutanoic acid | 0.8625 | 0.007312 |
| Orotidine | 0.8375 | 0.0096834 |
| Thyroxine | 0.81875 | 0.020645 |
| Heme | 0.8125 | 0.028791 |
| Pantetheine | 0.8125 | 0.029728 |
